# Supplementary material for: Synthesis of 6-Halo-Substituted Pericosine A and an Evaluation of Their Antitumor and Antiglycosidase Activities
Source: Mar Drugs. 2022 Jun 30;20(7):438. doi: 10.3390/md20070438 (PMC9323573; doi:10.3390/md20070438)
Supplement: Supplementary file 1 [file marinedrugs-20-00438-s001.zip › marinedrugs-1798432-supplementary.pdf]

Supporting Materials

Synthesis of 6-Halo-substituted Pericosine A and Their Evaluations for Antitumor and Antiglycosidase Activities

Yoshihide Usami[1,*], Yoshino Mizobuchi[1], Mai Ijuin,[1] Takeshi Yamada[2], Mizuki Morita[1], Koji Mizuki[1], Hiroki Yoneyama[1], and Shinya Harusawa[1]

Osaka University of Pharmaceutical Sciences, Nasahara 4-20-1, Takatsuki, Osaka 569-1094, Japan

Contents

1. ^{1}H- and ^{13}C-NMR spectra of new compounds and pericoxide, and HMBC spectrum of compound **12**

Figure S1. ^{1}H-NMR spectrum of (-)-**10**$_F$ in $CDCl_3$ (500MHz) page SM- 3
Figure S2. ^{13}C-NMR spectrum of (-)-**10**$_F$ in $CDCl_3$ (125 MHz) page SM- 4
Figure S3. ^{1}H-NMR spectrum of (+)-**10**$_F$ in $CDCl_3$ (400 MHz) page SM- 5
Figure S4. ^{13}C-NMR spectrum of (+)-**10**$_F$ in $CDCl_3$ (100 MHz) page SM- 5
Figure S5. ^{1}H-NMR spectrum of (-)-**1**$_F$ in acetone-d_6 (600 MHz) page SM- 6
Figure S6. ^{13}C-NMR spectrum of (-)-**1**$_F$ in acetone-d_6 (150 MHz) page SM- 7
Figure S7. ^{1}H-NMR spectrum of (+)-**1**$_F$ in acetone-d_6 (600 MHz) page SM- 8
Figure S8. ^{13}C-NMR spectrum of (+)-**1**$_F$ in acetone-d_6 (150 MHz) page SM- 9
Figure S9. ^{1}H-NMR spectrum of (-)-**10**$_{Br}$ in acetone-d_6 (600 MHz) page SM-10
Figure S10. ^{1}H-NMR spectrum of (-)-**10**$_{Br}$ in $CDCl_3$ (600 MHz) page SM-11
Figure S11. ^{13}C-NMR spectrum of (-)-**10**$_{Br}$ in acetone-d_6 (150 MHz) page SM-12
Figure S12. ^{1}H-NMR spectrum of (+)-**10**$_{Br}$ in acetone-d_6 (600 MHz) page SM-13
Figure S13. ^{13}C-NMR spectrum of (+)-**10**$_{Br}$ in acetone-d_6 (150 MHz) page SM-14
Figure S14. ^{1}H-NMR spectrum of (-)-**1**$_{Br}$ in methanol-d_4 (600 MHz) page SM-15
Figure S15. ^{13}C-NMR spectrum of (-)-**1**$_{Br}$ in methanol-d_4 (150 MHz) page SM-15
Figure S16. ^{1}H-NMR spectrum of (+)-**1**$_{Br}$ in methanol-d_4 (600 MHz) page SM-16
Figure S17. ^{13}C-NMR spectrum of (+)-**1**$_{Br}$ in methanol-d_4 (150 MHz) page SM-16
Figure S18. ^{1}H-NMR spectrum of (-)-**10**$_I$ in $CDCl_3$ (600 MHz) page SM-17
Figure S19. ^{13}C-NMR spectrum of (-)-**10**$_I$ in $CDCl_3$ (150 MHz) page SM-18
Figure S20. ^{1}H-NMR spectrum of (+)-**10**$_I$ in $CDCl_3$ (400 MHz) page SM-19
Figure S21. ^{13}C-NMR spectrum of (+)-**10**$_I$ in $CDCl_3$ (100 MHz) page SM-19
Figure S22. ^{1}H-NMR spectrum of (-)-**1**$_I$ in acetone-d_6 (600 MHz) page SM-20
Figure S23. ^{13}C-NMR spectrum of (-)-**1**$_I$ in acetone-d_6 (150 MHz) page SM-20
Figure S24. ^{1}H-NMR spectrum of (+)-**1**$_I$ in acetone-d_6 (600 MHz) page SM-21

Figure S25. ^{13}C-NMR spectrum of (+)-**1**$_{I}$ in acetone-d$_6$ (150 MHz) page SM-21
Figure S26. ^{1}H-NMR spectrum of (-)-**11** in methanol-d$_4$ (600 MHz) page SM-22
Figure S27. ^{13}C-NMR spectrum of (-)-**11** in methanol-d$_4$ (150 MHz) page SM-22
Figure S28. ^{1}H-NMR spectrum of (-)-**12** in methanol-d$_4$ (600 MHz) page SM-23
Figure S29. ^{13}C-NMR spectrum of (-)-**12** in methanol-d$_4$ (150 MHz) page SM-23
Figure S30. HMBC spectrum of (-)-**12** in methanol-d$_4$ (600 MHz) page SM-23
Figure S31. ^{1}H-NMR spectrum of (-)-**7** in methanol-d$_4$ (600 MHz) page SM-24
Figure S32. ^{13}C-NMR spectrum of (-)-**7** in methanol-d$_4$ (150 MHz) page SM-25
Figure S33. ^{1}H-NMR spectrum of (+)-**7** in methanol-d$_4$ (600 MHz) page SM-26
Figure S34. ^{13}C-NMR spectrum of (+)-**7** in methanol-d$_4$ (150 MHz) page SM-26

2. Table S1. Deprotection of (-)-**10**$_{Br}$ with Dowex® 50WX8-H page SM-27

1. ^{1}H- and ^{13}C-NMR spectra of new compounds and pericoxide

Figure S1. ^{1}H-NMR spectrum of (-)-**10F** in $CDCl_3$ (500 MHz)

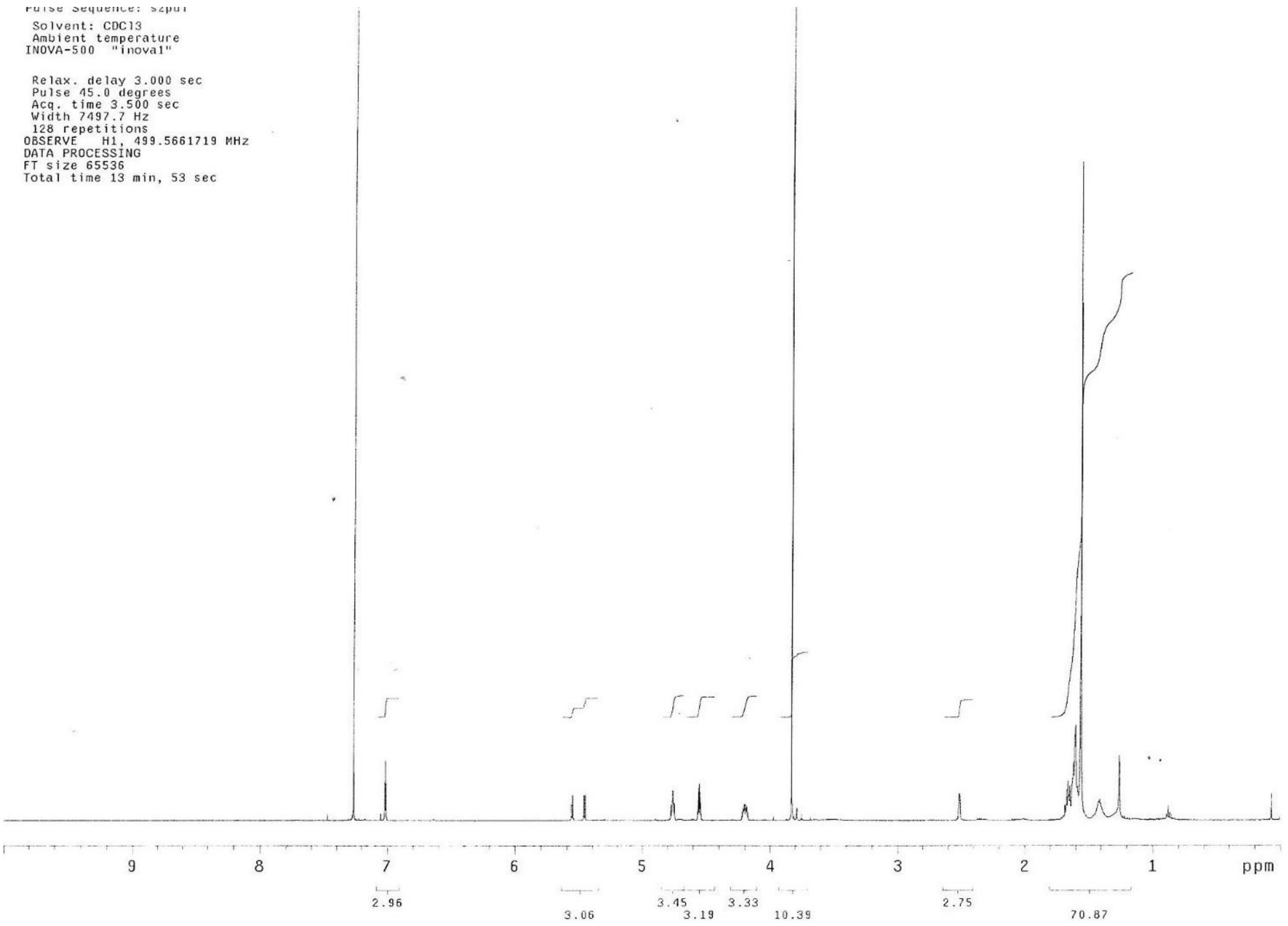

YF89-6

Fri Oct 8 08:24:59 JST 2010

INDEX	FREQUENCY(PPM)	Hz	HEIGHT
1	7.468	3730.9811	17.9
2	7.302	3647.6942	6.3
3	7.275	3634.4232	6.6
4	7.272	3633.0503	10.3
5	7.271	3632.1351	15.6
6	7.261	3627.5589	3492.0
7	7.256	3624.8132	11.8
8	7.253	3623.4403	7.1
9	7.222	3607.6524	6.5
10	7.050	3521.8485	18.0
11	7.020	3506.7470	106.0
12	7.014	3504.0013	166.5
13	7.009	3501.2556	103.4
14	5.563	2779.1302	70.1
15	5.551	2773.1811	71.0
16	5.552	2773.6387	71.7
17	5.467	2731.0800	70.4
18	5.456	2725.5885	72.5
19	5.300	2647.7930	6.5
20	4.775	2385.5764	39.9
21	4.773	2384.6612	41.9
22	4.769	2382.3730	43.7
23	4.767	2381.4578	46.4
24	4.762	2379.1697	84.0
25	4.756	2376.1952	82.9
26	4.752	2373.9071	48.9
27	4.750	2372.9918	45.0
28	4.745	2370.7037	48.6
29	4.744	2369.7885	43.6
30	4.566	2281.2389	54.4
31	4.560	2278.0355	89.4
32	4.554	2275.0610	102.7
33	4.548	2271.8577	79.2
34	4.542	2268.8831	49.1
35	4.223	2109.8599	24.5
36	4.216	2106.1990	39.9
37	4.212	2104.3685	33.1
38	4.208	2102.3092	31.7
39	4.205	2100.4787	45.3
40	4.195	2095.6737	44.9
41	4.191	2093.8432	32.3
42	4.187	2091.7839	32.3
43	4.184	2090.1823	39.0
44	4.176	2086.2925	22.9
45	3.973	1984.9295	11.0
46	3.828	1912.3966	2278.9
47	3.810	1903.4730	18.9
48	3.790	1893.4054	35.2
49	3.783	1889.7444	36.3
50	3.749	1872.8124	17.9
51	3.678	1837.3468	12.4
52	2.517	1257.5415	70.3
53	2.514	1255.9398	75.1
54	2.509	1253.4229	74.1
55	2.506	1251.8212	68.2
56	2.351	1174.7121	8.6
57	2.336	1167.1614	6.0
58	1.710	854.3776	8.0
59	1.695	846.8269	12.0
60	1.684	841.3355	43.4
61	1.669	834.0135	85.7
62	1.660	829.4373	109.2
63	1.653	826.0052	72.9
64	1.646	822.1154	96.1
65	1.631	814.5646	89.3
66	1.626	812.2765	115.7
67	1.615	806.7851	163.2
68	1.605	801.9801	258.1
69	1.600	799.2344	266.8
70	1.590	794.2005	94.6
71	1.587	792.5989	94.2
72	1.560	779.3279	1835.9
73	1.520	759.4214	15.8
74	1.458	728.5320	14.6
75	1.420	709.5407	53.0
76	1.415	707.0238	51.4
77	1.410	704.2781	59.6
78	1.401	699.7019	47.7
79	1.379	688.7190	20.9
80	1.255	627.1690	181.1
81	1.231	614.8132	13.6
82	1.223	610.9235	8.8
83	1.217	607.9489	11.3
84	1.195	596.7372	7.0
85	1.193	595.8220	7.0
86	1.181	589.8729	6.0
87	1.178	588.7288	5.9
88	0.971	485.3066	6.2
89	0.956	477.5270	6.7
90	0.895	446.8664	17.3
91	0.881	440.0021	42.2
92	0.868	433.8243	21.9
93	0.855	427.1888	17.4
94	0.844	421.6973	6.0
95	0.070	34.7790	76.3
96	0.007	3.4320	9.3
97	-0.000	-0.0001	326.4
98	-0.006	-3.2035	8.1

Figure S2. ^{13}C-NMR spectrum of (-)-**10F** in $CDCl_3$ (125 MHz)

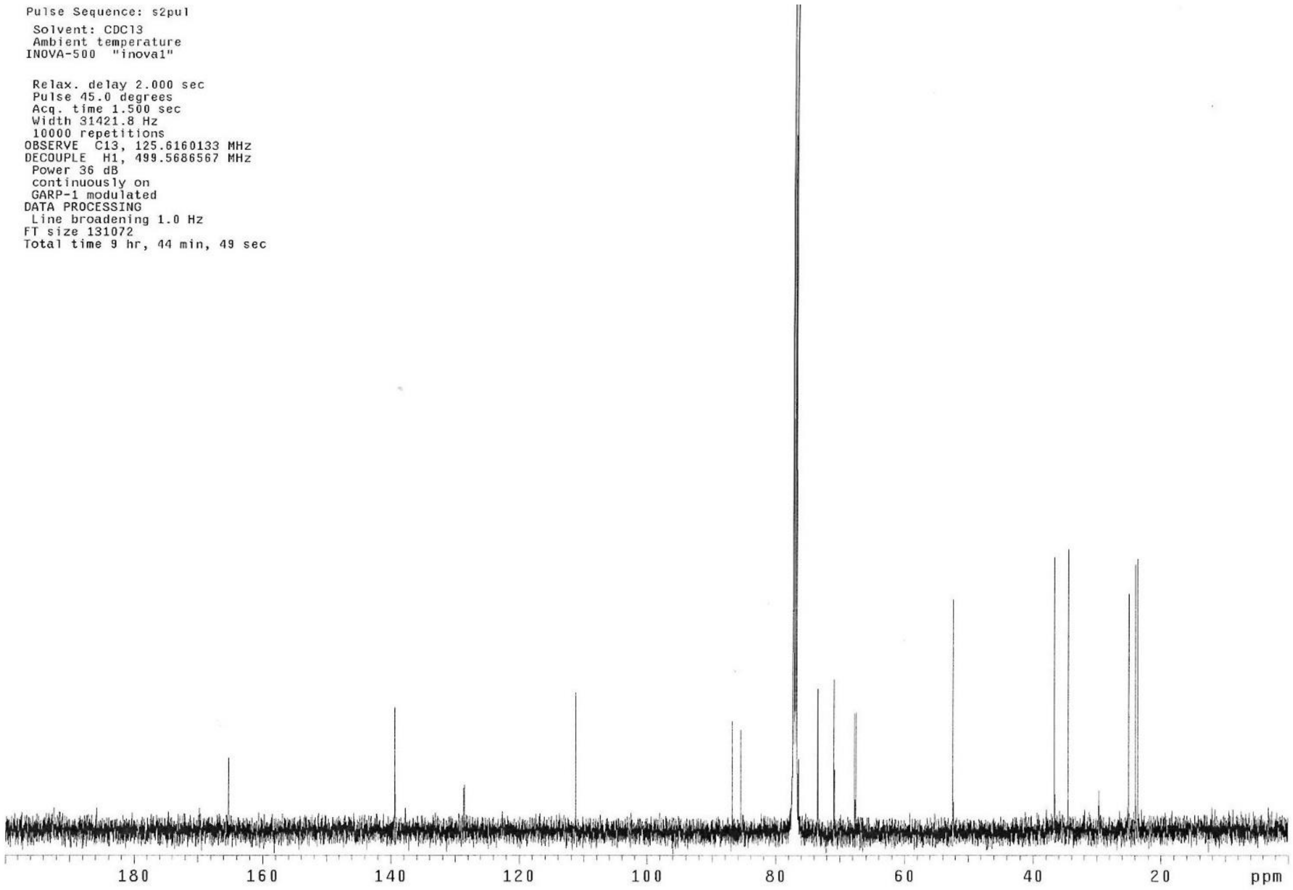

INDEX	FREQUENCY(PPM)	Hz	HEIGHT
1	165.228	20757.5297	13.9
2	139.402	17513.0293	23.5
3	139.356	17507.2758	22.6
4	128.659	16163.3517	8.0
5	128.517	16145.6117	8.8
6	111.229	13973.6615	26.4
7	86.891	10916.1502	20.9
8	85.548	10747.3806	19.3
9	77.247	9704.5568	1377.7
10	76.992	9672.4330	1423.1
11	76.736	9640.3093	1377.9
12	76.419	9600.5141	13.7
13	73.408	9222.2208	27.1
14	73.362	9216.4673	27.1
15	70.885	8905.2983	26.1
16	70.866	8902.9010	29.0
17	67.687	8503.5115	22.5
18	67.489	8478.5796	22.6
19	52.364	6578.4828	44.3
20	36.629	4601.6725	52.5
21	34.495	4333.6548	54.0
22	29.691	3730.0157	7.6
23	24.928	3131.6506	45.4
24	23.893	3001.7172	51.0
25	23.561	2960.0042	52.1

Figure S3. ^{1}H-NMR spectrum of (+)-**10F** in $CDCl_3$ (400 MHz)

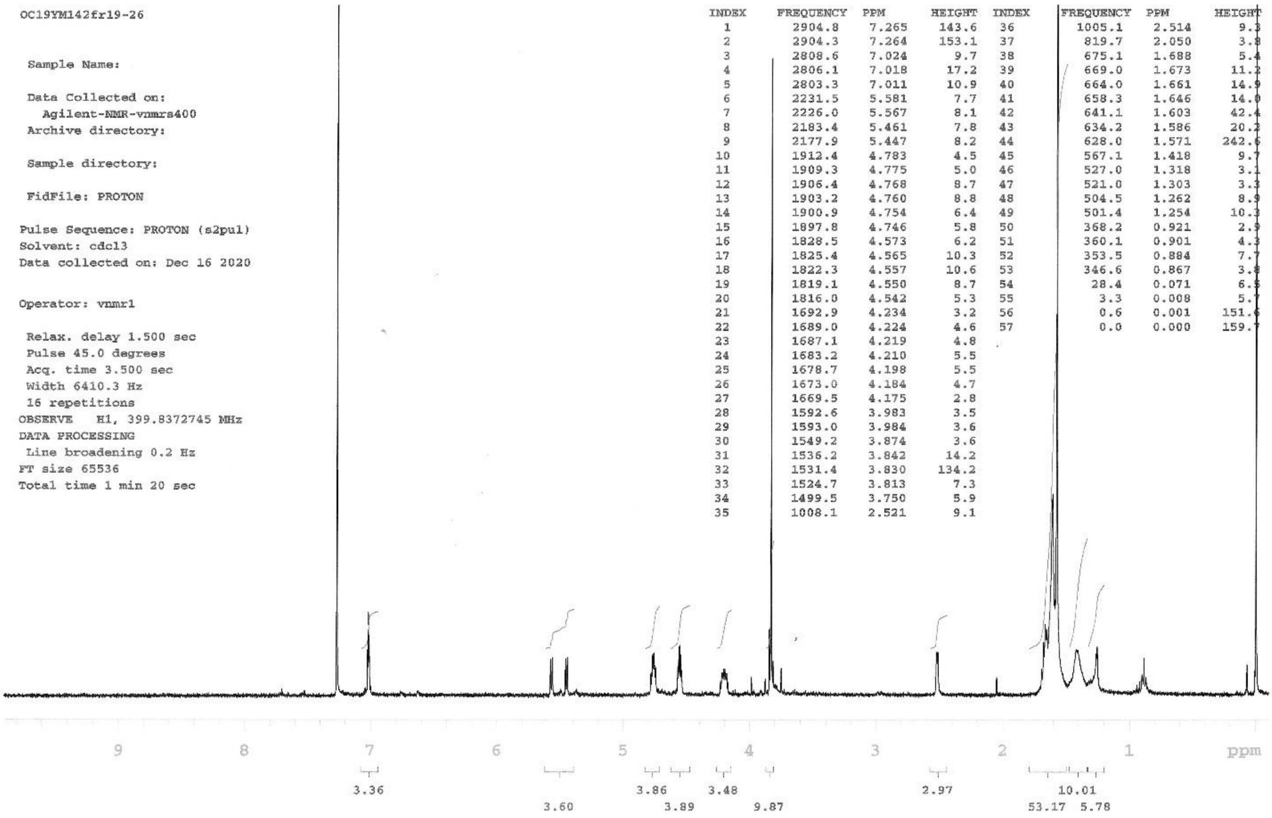

OC19YM142fr19-26

Sample Name:

Data Collected on:
Agilent-NMR-vnmrs400
Archive directory:

Sample directory:

FidFile: PROTON

Pulse Sequence: PROTON (s2pul)
Solvent: cdcl3
Data collected on: Dec 16 2020

Operator: vnmr1

Relax. delay 1.500 sec
Pulse 45.0 degrees
Acq. time 3.500 sec
Width 6410.3 Hz
16 repetitions
OBSERVE H1, 399.8372745 MHz
DATA PROCESSING
Line broadening 0.2 Hz
FT size 65536
Total time 1 min 20 sec

INDEX	FREQUENCY	PPM	HEIGHT	INDEX	FREQUENCY	PPM	HEIGHT
1	2904.8	7.265	143.6	36	1005.1	2.514	9.3
2	2904.3	7.264	153.1	37	819.7	2.050	3.8
3	2808.6	7.024	9.7	38	675.1	1.688	5.4
4	2806.1	7.018	17.2	39	669.0	1.673	11.2
5	2803.3	7.011	10.9	40	664.0	1.661	14.9
6	2231.5	5.581	7.7	41	658.3	1.646	14.0
7	2226.0	5.567	8.1	42	641.1	1.603	42.4
8	2183.4	5.461	7.8	43	634.2	1.586	20.2
9	2177.9	5.447	8.2	44	628.0	1.571	242.6
10	1912.4	4.783	4.5	45	567.1	1.418	9.7
11	1909.3	4.775	5.0	46	527.0	1.318	3.1
12	1906.4	4.768	8.7	47	521.0	1.303	3.3
13	1903.2	4.760	8.8	48	504.5	1.262	8.9
14	1900.9	4.754	6.4	49	501.4	1.254	10.3
15	1897.8	4.746	5.8	50	368.2	0.921	2.9
16	1828.5	4.573	6.2	51	360.1	0.901	4.3
17	1825.4	4.565	10.3	52	353.5	0.884	7.7
18	1822.3	4.557	10.6	53	346.6	0.867	3.8
19	1819.1	4.550	8.7	54	28.4	0.071	6.5
20	1816.0	4.542	5.3	55	3.3	0.008	5.7
21	1692.9	4.234	3.2	56	0.6	0.001	151.6
22	1689.0	4.224	4.6	57	0.0	0.000	159.7
23	1687.1	4.219	4.8				
24	1683.2	4.210	5.5				
25	1678.7	4.198	5.5				
26	1673.0	4.184	4.7				
27	1669.5	4.175	2.8				
28	1592.6	3.983	3.5				
29	1593.0	3.984	3.6				
30	1549.2	3.874	3.6				
31	1536.2	3.842	14.2				
32	1531.4	3.830	134.2				
33	1524.7	3.813	7.3				
34	1499.5	3.750	5.9				
35	1008.1	2.521	9.1				

Figure S4. ^{13}C-NMR spectrum of (+)-**10F** in $CDCl_3$ (100 MHz)

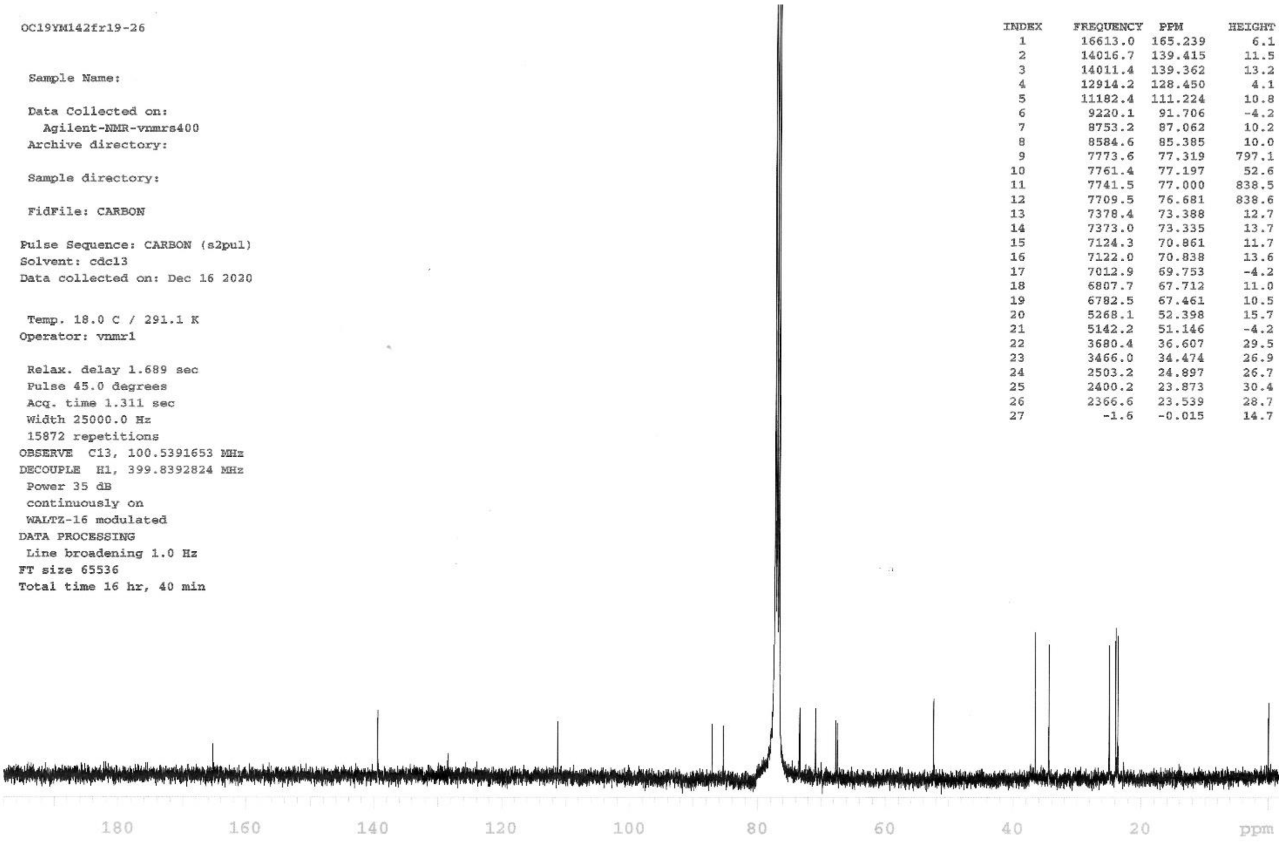

OC19YM142fr19-26

Sample Name:

Data Collected on:
Agilent-NMR-vnmrs400
Archive directory:

Sample directory:

FidFile: CARBON

Pulse Sequence: CARBON (s2pul)
Solvent: cdcl3
Data collected on: Dec 16 2020

Temp. 18.0 C / 291.1 K
Operator: vnmr1

Relax. delay 1.689 sec
Pulse 45.0 degrees
Acq. time 1.311 sec
Width 25000.0 Hz
15872 repetitions
OBSERVE C13, 100.5391653 MHz
DECOUPLE H1, 399.8392824 MHz
Power 35 dB
continuously on
WALTZ-16 modulated
DATA PROCESSING
Line broadening 1.0 Hz
FT size 65536
Total time 16 hr, 40 min

INDEX	FREQUENCY	PPM	HEIGHT
1	16613.0	165.239	6.1
2	14016.7	139.415	11.5
3	14011.4	139.362	13.2
4	12914.2	128.450	4.1
5	11182.4	111.224	10.8
6	9220.1	91.706	-4.2
7	8753.2	87.062	10.2
8	8584.6	85.385	10.0
9	7773.6	77.319	797.1
10	7761.4	77.197	52.6
11	7741.5	77.000	838.5
12	7709.5	76.681	838.6
13	7378.4	73.388	12.7
14	7373.0	73.335	13.7
15	7124.3	70.861	11.7
16	7122.0	70.838	13.6
17	7012.9	69.753	-4.2
18	6807.7	67.712	11.0
19	6782.5	67.461	10.5
20	5268.1	52.398	15.7
21	5142.2	51.146	-4.2
22	3680.4	36.607	29.5
23	3466.0	34.474	26.9
24	2503.2	24.897	26.7
25	2400.2	23.873	30.4
26	2366.6	23.539	28.7
27	-1.6	-0.015	14.7

Figure S5. ^{1}H-NMR spectrum of (-)-**1**$_F$ in acetone-d$_6$ (600 MHz)

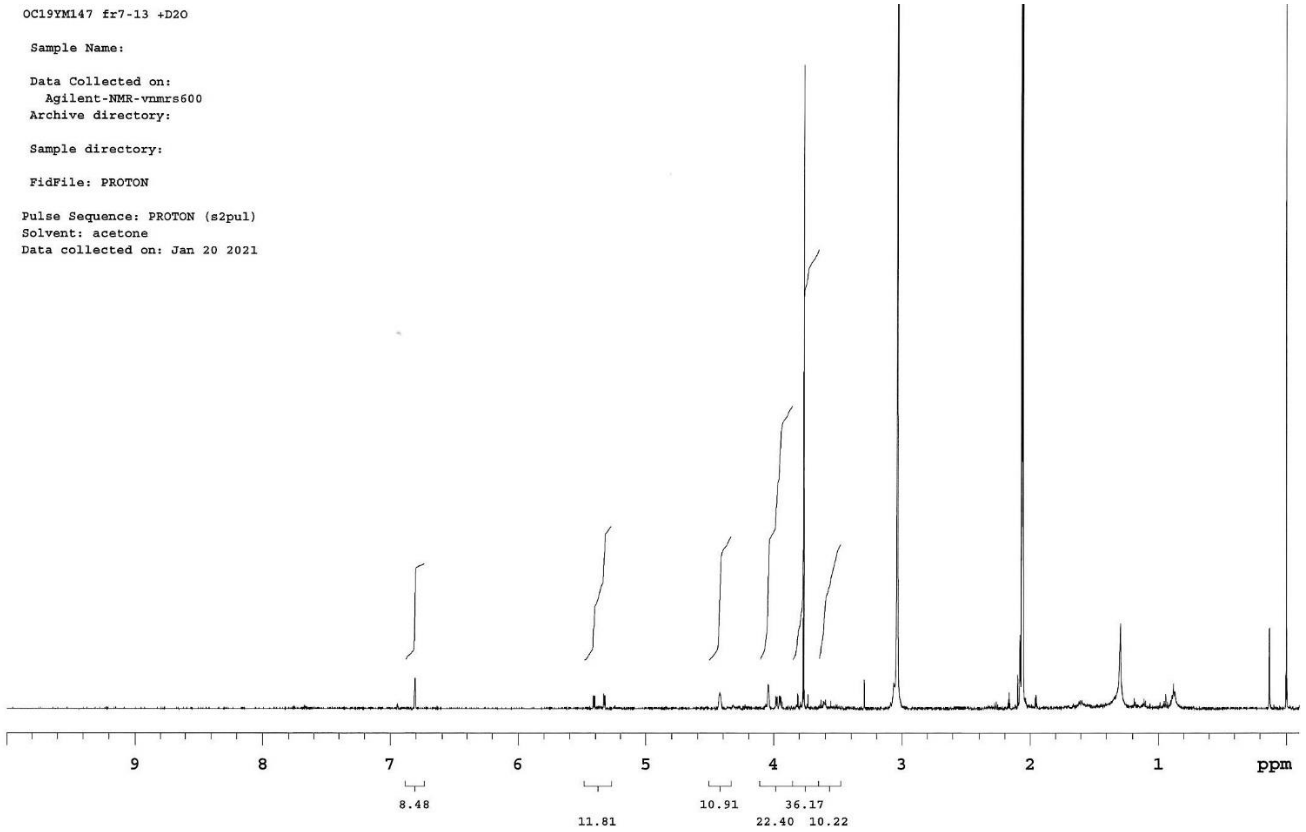

OC19YM147 fr7-13 +D2O

INDEX	FREQUENCY	PPM	HEIGHT	INDEX	FREQUENCY	PPM	HEIGHT	INDEX	FREQUENCY	PPM	HEIGHT
1	4166.8	6.946	7.0	51	1978.9	3.299	51.1	101	775.3	1.292	146.8
2	4166.2	6.945	7.2	52	1853.9	3.090	8.8	102	711.9	1.187	18.3
3	4086.1	6.811	48.8	53	1839.6	3.066	45.4	103	707.5	1.179	12.6
4	4085.2	6.810	53.6	54	1823.4	3.040	2108.2	104	674.9	1.125	7.3
5	4084.4	6.808	52.9	55	1821.7	3.037	2143.0	105	666.4	1.111	18.2
6	4083.5	6.807	45.3	56	1807.6	3.013	17.3	106	660.2	1.101	14.7
7	3249.8	5.417	22.0	57	1367.7	2.280	8.0	107	639.7	1.066	7.2
8	3249.2	5.416	23.1	58	1360.4	2.268	12.7	108	592.7	0.988	9.1
9	3248.1	5.414	23.1	59	1353.0	2.255	7.4	109	575.7	0.960	9.2
10	3247.5	5.413	22.1	60	1304.3	2.174	9.9	110	574.3	0.957	8.0
11	3243.1	5.406	22.7	61	1302.3	2.171	16.4	111	572.8	0.955	13.6
12	3241.9	5.404	22.3	62	1299.9	2.167	28.7	112	566.9	0.945	9.2
13	3241.3	5.403	21.3	63	1297.9	2.163	17.0	113	565.2	0.942	25.5
14	3200.2	5.335	26.3	64	1295.5	2.160	10.5	114	557.8	0.930	12.5
15	3199.7	5.334	24.7	65	1270.3	2.118	8.2	115	550.2	0.917	8.5
16	3198.5	5.332	24.2	66	1259.7	2.100	59.0	116	543.2	0.905	14.7
17	3194.1	5.324	23.2	67	1258.6	2.098	55.3	117	535.5	0.893	23.8
18	3193.5	5.323	23.2	68	1258.0	2.097	51.9	118	528.2	0.880	43.7
19	3192.3	5.321	23.3	69	1255.9	2.094	8.1	119	524.7	0.875	29.7
20	2656.8	4.429	26.9	70	1250.0	2.084	118.2	120	521.1	0.869	28.4
21	2654.7	4.425	26.6	71	1248.9	2.082	58.6	121	518.5	0.864	21.1
22	2437.6	4.063	7.1	72	1248.0	2.080	127.5	122	78.1	0.130	139.2
23	2434.4	4.058	7.2	73	1245.6	2.076	113.3	123	76.3	0.127	21.3
24	2426.4	4.045	43.5	74	1242.4	2.071	1707.9	124	75.4	0.126	17.8
25	2425.3	4.043	37.4	75	1240.1	2.067	3063.9	125	74.2	0.124	9.4
26	2424.1	4.041	41.8	76	1238.0	2.064	5056.4	126	58.1	0.097	7.5
27	2390.9	3.986	21.8	77	1235.7	2.060	3093.8	127	3.2	0.005	57.9
28	2388.9	3.982	19.3	78	1233.6	2.056	1683.8	128	0.0	0.000	1997.6
29	2384.8	3.975	21.4	79	1230.4	2.051	32.2	129	-3.5	-0.006	53.1
30	2382.7	3.972	19.8	80	1224.5	2.041	18.5				
31	2374.2	3.958	22.9	81	1222.2	2.037	18.5				
32	2372.2	3.954	21.4	82	1220.1	2.034	13.4				
33	2368.0	3.947	22.2	83	1217.8	2.030	10.0				
34	2366.3	3.944	20.3	84	1178.4	1.964	9.4				
35	2360.1	3.934	13.0	85	1176.1	1.960	19.9				
36	2288.5	3.815	26.0	86	1173.8	1.957	24.5				
37	2285.3	3.809	23.8	87	1171.7	1.953	21.2				
38	2275.6	3.793	9.3	88	1169.4	1.949	10.1				
39	2274.4	3.791	12.9	89	995.9	1.660	7.0				
40	2273.3	3.789	10.1	90	968.6	1.615	11.7				
41	2265.9	3.777	23.3	91	964.2	1.607	13.0				
42	2263.9	3.774	156.4	92	956.0	1.594	14.4				
43	2259.2	3.766	1102.7	93	950.4	1.584	10.2				
44	2239.5	3.733	24.9	94	944.3	1.574	7.9				
45	2184.1	3.641	8.6	95	930.5	1.551	7.5				
46	2179.1	3.632	16.0	96	925.2	1.542	7.4				
47	2168.5	3.615	13.7	97	853.3	1.422	8.0				
48	2165.6	3.610	12.5	98	846.0	1.410	8.1				
49	2158.8	3.599	15.9	99	839.5	1.399	8.5				
50	2133.6	3.557	11.6	100	832.2	1.387	9.5				

Figure S6. ^{13}C-NMR spectrum of (-)-**1**$_F$ in acetone-d_6 (150 MHz)

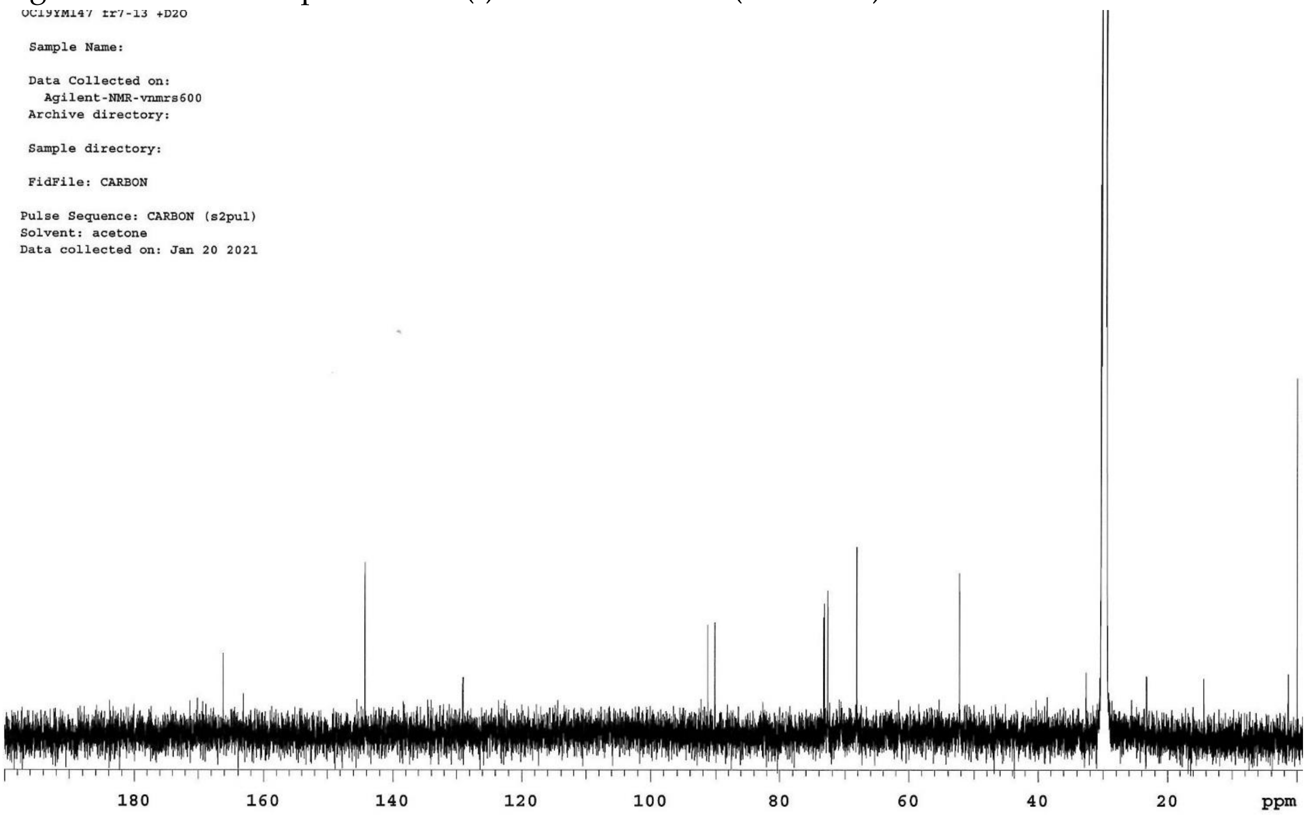

OC19YM147 fr7-13 +D2O

INDEX	FREQUENCY	PPM	HEIGHT
1	25081.1	166.271	18.8
2	24611.7	163.160	10.6
3	21765.7	144.293	35.1
4	21758.8	144.247	37.6
5	19479.2	129.134	12.6
6	19460.7	129.012	14.0
7	13765.3	91.255	24.7
8	13597.7	90.144	25.2
9	11059.2	73.315	26.3
10	11038.3	73.177	29.1
11	10966.7	72.702	31.8
12	10960.9	72.664	28.4
13	10287.0	68.196	40.8
14	7880.2	52.241	35.4
15	4921.0	32.623	14.9
16	4582.3	30.377	115.3
17	4563.8	30.255	2878.2
18	4544.1	30.124	8497.2
19	4524.5	29.994	13852.0
20	4506.0	29.872	16817.6
21	4486.3	29.741	17205.7
22	4466.7	29.611	8542.2
23	4447.0	29.481	2339.7
24	4425.0	29.335	24.6
25	3516.5	23.312	14.2
26	2165.1	14.353	13.7
27	211.5	1.402	14.6
28	-0.0	-0.000	75.5

Figure S7. ^{1}H-NMR spectrum of (+)-**1**$_{F}$ in acetone-d$_6$ (600 MHz)

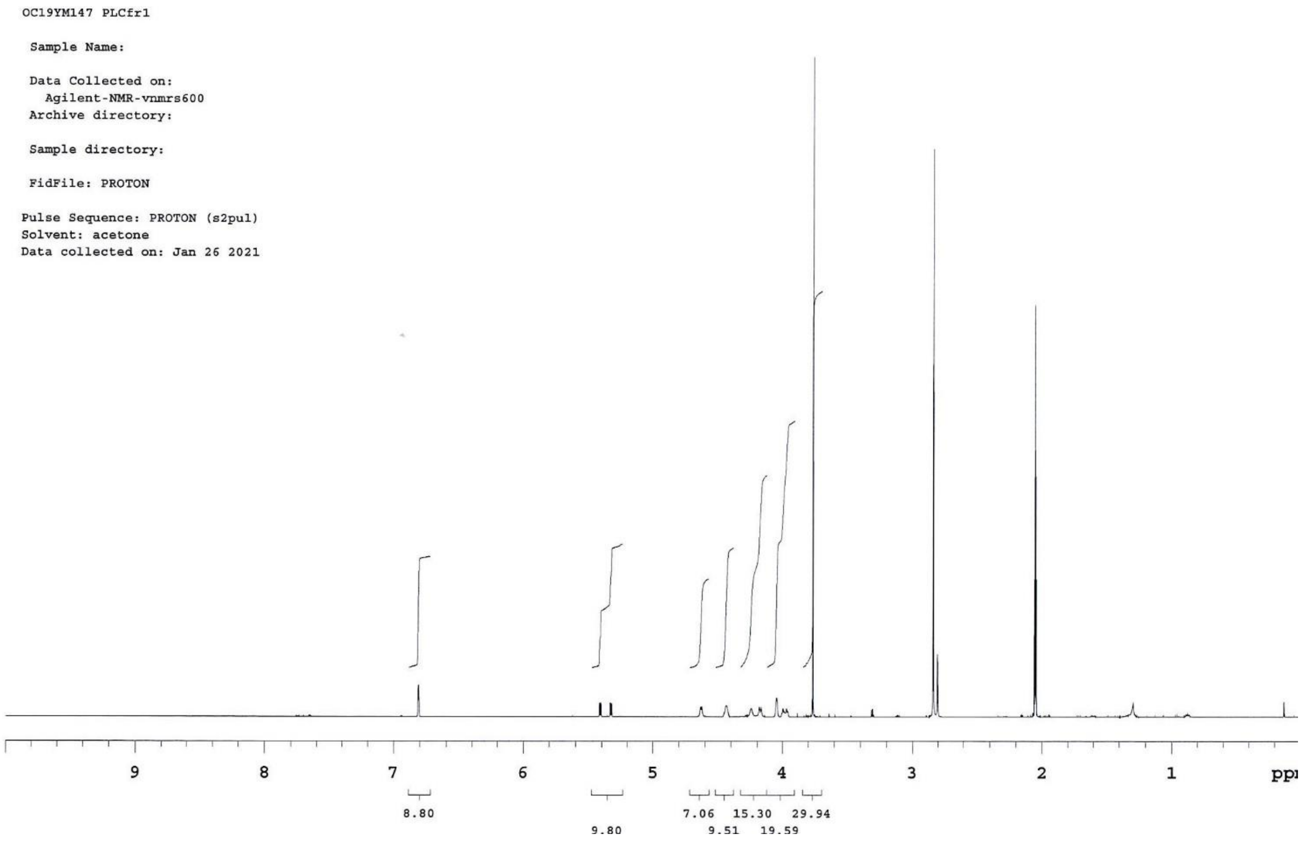

OC19YM147 PLCfr1

NDEX	FREQUENCY	PPM	HEIGHT	INDEX	FREQUENCY	PPM	HEIGHT
1	4088.5	6.815	31.0	51	1236.5	2.061	222.9
2	4087.6	6.814	46.0	52	1234.2	2.057	388.9
3	4086.7	6.812	51.5	53	1232.1	2.054	669.1
4	4085.8	6.811	52.1	54	1229.8	2.050	390.1
5	4084.7	6.809	43.0	55	1227.7	2.047	223.1
6	4084.1	6.808	31.2	56	775.6	1.293	23.2
7	4082.9	6.806	22.2	57	528.2	0.880	5.8
8	3249.8	5.417	21.8	58	78.1	0.130	24.4
9	3249.2	5.416	22.4	59	3.2	0.005	7.9
10	3248.1	5.414	22.8	60	0.0	0.000	262.0
11	3247.5	5.413	21.9	61	-3.2	-0.005	5.7
12	3243.7	5.407	21.1				
13	3243.1	5.406	22.0				
14	3241.9	5.404	21.2				
15	3241.3	5.403	20.4				
16	3200.2	5.335	22.8				
17	3199.7	5.334	22.7				
18	3198.5	5.332	23.3				
19	3197.9	5.331	22.1				
20	3194.1	5.324	21.4				
21	3193.5	5.323	22.2				
22	3192.3	5.321	21.5				
23	3191.7	5.320	20.7				
24	2779.7	4.634	16.0				
25	2773.9	4.624	16.3				
26	2661.5	4.437	18.6				
27	2546.5	4.245	13.9				
28	2547.0	4.246	13.9				
29	2509.2	4.183	15.9				
30	2501.0	4.169	14.2				
31	2427.9	4.047	30.7				
32	2398.6	3.998	13.1				
33	2396.8	3.995	13.0				
34	2392.7	3.988	9.4				
35	2385.9	3.977	6.7				
36	2382.1	3.971	13.2				
37	2380.4	3.968	13.0				
38	2376.3	3.961	9.1				
39	2330.2	3.884	6.1				
40	2262.4	3.771	29.2				
41	2258.3	3.764	1070.1				
42	2250.7	3.752	4.3				
43	2183.2	3.639	5.7				
44	1990.1	3.317	11.9				
45	1984.8	3.309	13.1				
46	1703.1	2.839	921.5				
47	1682.9	2.805	101.7				
48	1244.2	2.074	6.4				
49	1242.1	2.071	7.2				
50	1239.8	2.067	6.4				

Figure S8. ^{13}C-NMR spectrum of (+)-**1**$_F$ in acetone-d$_6$ (150 MHz)

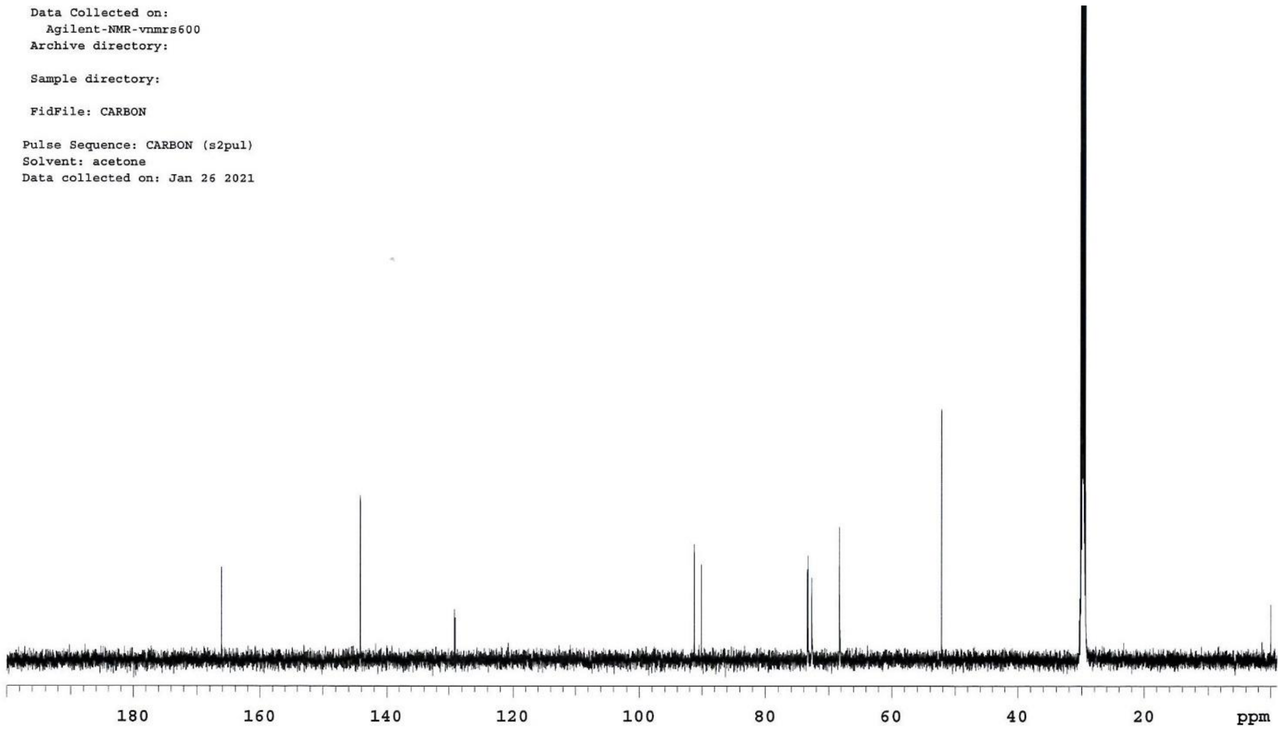

INDEX	FREQUENCY	PPM	HEIGHT
1	25068.3	166.187	19.9
2	21753.0	144.208	34.9
3	21746.1	144.162	33.6
4	19483.9	129.165	11.0
5	19465.4	129.043	9.2
6	13764.1	91.247	24.7
7	13596.5	90.136	20.5
8	11081.1	73.461	19.4
9	11060.3	73.323	22.3
10	10970.1	72.725	17.6
11	10300.8	68.288	28.1
12	10298.5	68.272	28.3
13	10283.5	68.173	6.6
14	7874.5	52.202	53.2
15	4578.8	30.354	13.3
16	4560.3	30.232	444.8
17	4540.6	30.101	1385.7
18	4521.0	29.971	2346.9
19	4502.5	29.849	2554.4
20	4482.8	29.718	2720.0
21	4463.2	29.588	1387.5
22	4443.5	29.458	386.4
23	-0.0	-0.000	12.1

Figure S9. ^{1}H-NMR spectrum of (-)-**10$_{Br}$** in acetone-d$_6$ (600 MHz)

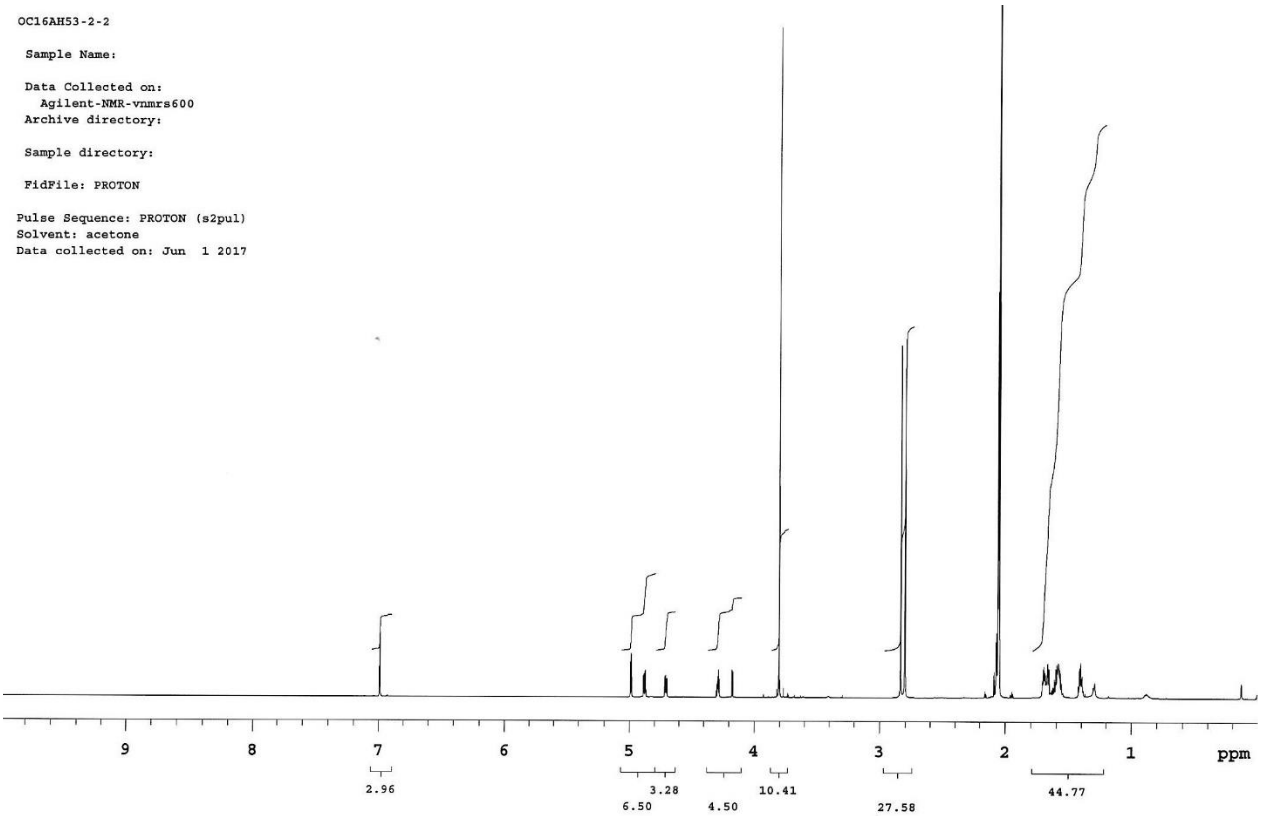

OC16AH53-2-2

INDEX	FREQUENCY	PPM	HEIGHT	INDEX	FREQUENCY	PPM	HEIGHT
1	4196.2	6.995	92.4	51	1227.7	2.047	1201.9
2	4194.7	6.992	167.2	52	1224.5	2.041	29.3
3	4192.9	6.989	100.9	53	1026.2	1.711	54.9
4	4191.5	6.987	197.1	54	1020.3	1.701	97.1
5	2993.1	4.989	134.6	55	1014.4	1.691	78.0
6	2989.3	4.983	120.1	56	1009.7	1.683	53.9
7	2988.7	4.982	130.5	57	1001.8	1.670	104.9
8	2932.0	4.888	67.2	58	996.5	1.661	89.1
9	2929.4	4.883	72.2	59	983.0	1.639	25.3
10	2928.8	4.882	68.9	60	977.1	1.629	33.4
11	2925.3	4.876	79.5	61	970.1	1.617	57.8
12	2922.4	4.871	83.3	62	964.2	1.607	90.0
13	2921.8	4.870	79.1	63	962.2	1.604	46.5
14	2829.3	4.716	39.1	64	958.4	1.598	101.7
15	2828.5	4.715	63.9	65	956.0	1.594	88.8
16	2825.8	4.710	41.8	66	951.9	1.587	106.2
17	2824.9	4.709	68.3	67	950.4	1.584	102.5
18	2822.3	4.705	36.5	68	946.6	1.578	73.4
19	2821.4	4.703	58.0	69	943.4	1.573	85.5
20	2818.8	4.699	35.7	70	940.2	1.567	35.0
21	2817.9	4.697	58.7	71	937.8	1.563	47.3
22	2574.0	4.291	49.8	72	855.4	1.426	37.4
23	2572.0	4.287	63.7	73	849.5	1.416	92.1
24	2570.5	4.285	55.5	74	843.6	1.406	108.6
25	2568.2	4.281	84.9	75	837.8	1.397	67.7
26	2566.1	4.278	33.6	76	832.2	1.387	22.6
27	2564.1	4.274	57.1	77	783.8	1.307	32.6
28	2503.9	4.174	85.4	78	775.6	1.293	47.4
29	2499.8	4.167	79.1	79	78.9	0.132	45.9
30	2292.9	3.822	25.6	80	0.0	0.000	570.0
31	2283.2	3.806	2040.0				
32	2279.1	3.799	55.0				
33	2277.4	3.796	22.7				
34	2260.6	3.768	29.1				
35	1701.6	2.837	1071.5				
36	1682.3	2.804	752.2				
37	1681.4	2.803	930.2				
38	1253.9	2.090	70.6				
39	1253.0	2.089	77.5				
40	1252.1	2.087	79.9				
41	1245.4	2.076	90.4				
42	1244.5	2.074	170.4				
43	1242.1	2.071	197.4				
44	1241.0	2.069	100.9				
45	1240.1	2.067	181.3				
46	1239.2	2.066	77.4				
47	1236.5	2.061	1234.5				
48	1234.5	2.058	2176.6				
49	1232.1	2.054	3577.8				
50	1230.1	2.051	2143.0				

Figure S10. ¹H-NMR spectrum of (-)-**10Br** in $CDCl_3$ (600 MHz)

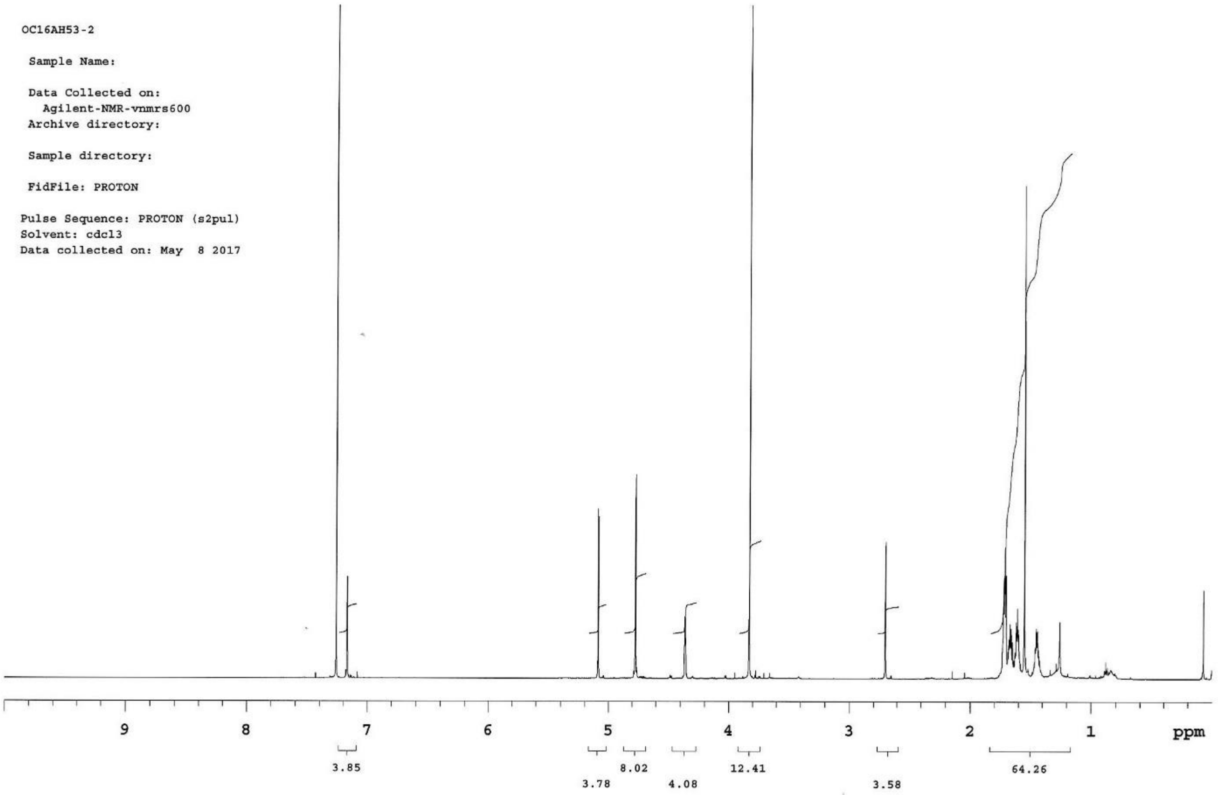

OC16AH53-2

INDEX	FREQUENCY	PPM	HEIGHT
1	4355.8	7.261	735.3
2	4302.7	7.172	81.5
3	4301.8	7.171	78.4
4	4300.9	7.169	86.1
5	4299.8	7.167	81.4
6	3053.8	5.091	143.1
7	3050.0	5.084	136.3
8	2864.8	4.776	152.0
9	2862.8	4.772	169.0
10	2861.3	4.770	172.1
11	2620.1	4.368	21.2
12	2617.8	4.364	41.3
13	2616.3	4.361	52.6
14	2615.4	4.360	40.1
15	2614.2	4.358	51.2
16	2612.8	4.355	37.8
17	2610.4	4.351	20.4
18	2299.1	3.832	50.3
19	2296.4	3.828	1140.3
20	1621.2	2.703	111.7
21	1618.9	2.699	115.3
22	1034.1	1.724	85.5
23	1029.1	1.715	109.5
24	1022.9	1.705	87.7
25	1013.5	1.690	23.2
26	1008.0	1.680	33.6
27	1002.7	1.671	46.4
28	997.7	1.663	42.5
29	991.5	1.653	31.3
30	985.4	1.643	16.0
31	978.9	1.632	22.4
32	972.7	1.622	48.0
33	966.9	1.612	59.2
34	961.3	1.602	42.0
35	955.7	1.593	18.5
36	953.4	1.589	15.2
37	930.5	1.551	414.7
38	880.9	1.468	13.4
39	875.0	1.459	26.7
40	869.8	1.450	42.5
41	864.5	1.441	40.5
42	858.6	1.431	24.4
43	853.3	1.422	15.9
44	770.6	1.285	13.5
45	752.4	1.254	48.2
46	528.2	0.880	14.5
47	41.4	0.069	75.1
48	0.0	0.000	252.3

Figure S11. ^{13}C-NMR spectrum of (-)-**10**$_{Br}$ in acetone-d$_6$ (150 MHz)

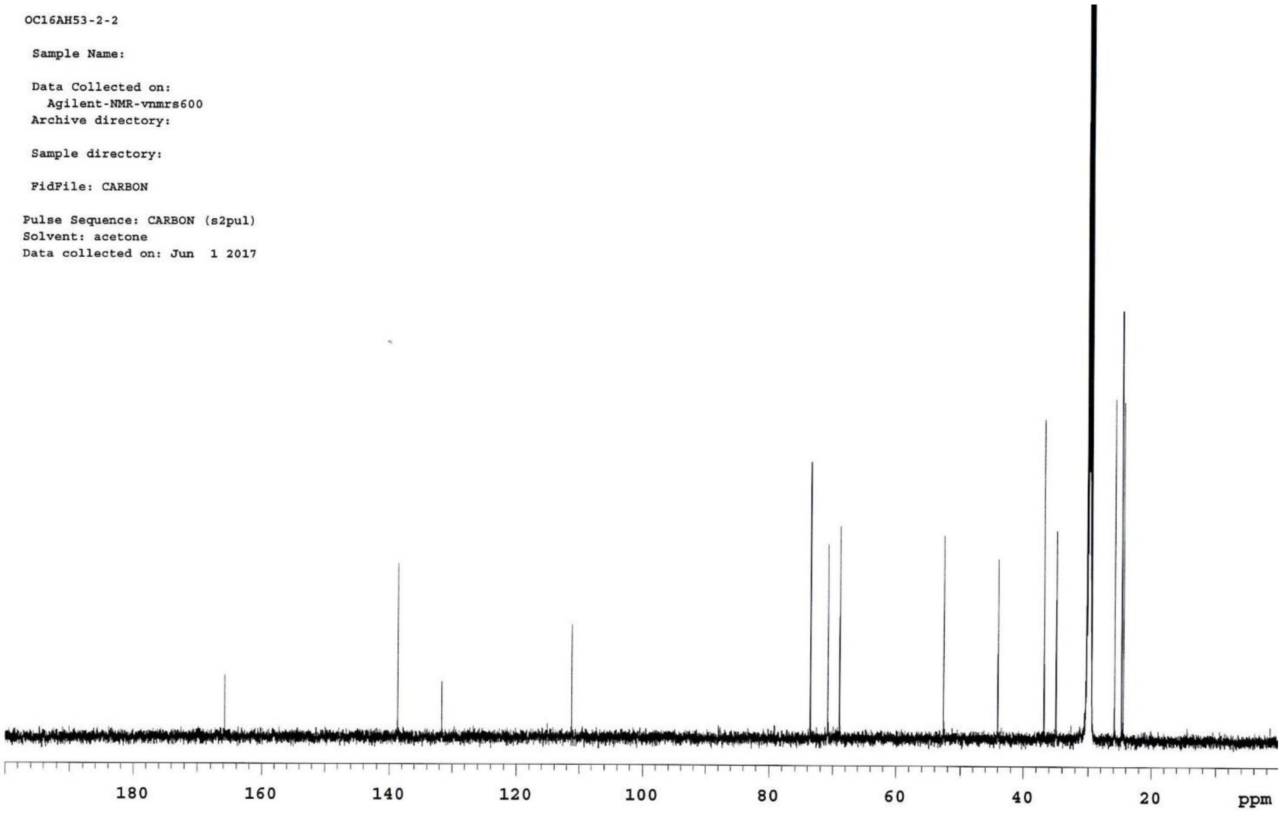

OC16AH53-2-2

INDEX	FREQUENCY	PPM	HEIGHT
1	31119.8	206.304	77.1
2	31100.2	206.174	4823.7
3	31080.5	206.044	63.0
4	25007.1	165.780	13.4
5	20914.9	138.652	36.8
6	19850.3	131.594	12.2
7	16763.9	111.133	24.1
8	11097.3	73.568	58.5
9	10680.0	70.801	41.2
10	10403.7	68.970	26.0
11	10392.2	68.893	45.0
12	7936.9	52.616	43.1
13	6639.9	44.018	38.3
14	5549.8	36.792	67.5
15	5268.9	34.929	44.2
16	5266.6	34.914	33.0
17	4614.6	30.592	6.2
18	4578.8	30.354	28.6
19	4560.3	30.232	501.5
20	4540.6	30.101	1662.8
21	4521.0	29.971	3020.1
22	4502.5	29.849	2690.9
23	4482.8	29.718	3021.6
24	4463.2	29.588	1659.6
25	4443.5	29.458	496.2
26	3897.9	25.841	71.7
27	3728.0	24.714	90.3
28	3685.2	24.431	71.0
29	-0.0	-0.000	10.8

Figure S12. ^{1}H-NMR spectrum of (+)-**10**$_{Br}$ in acetone-d$_6$ (600 MHz)

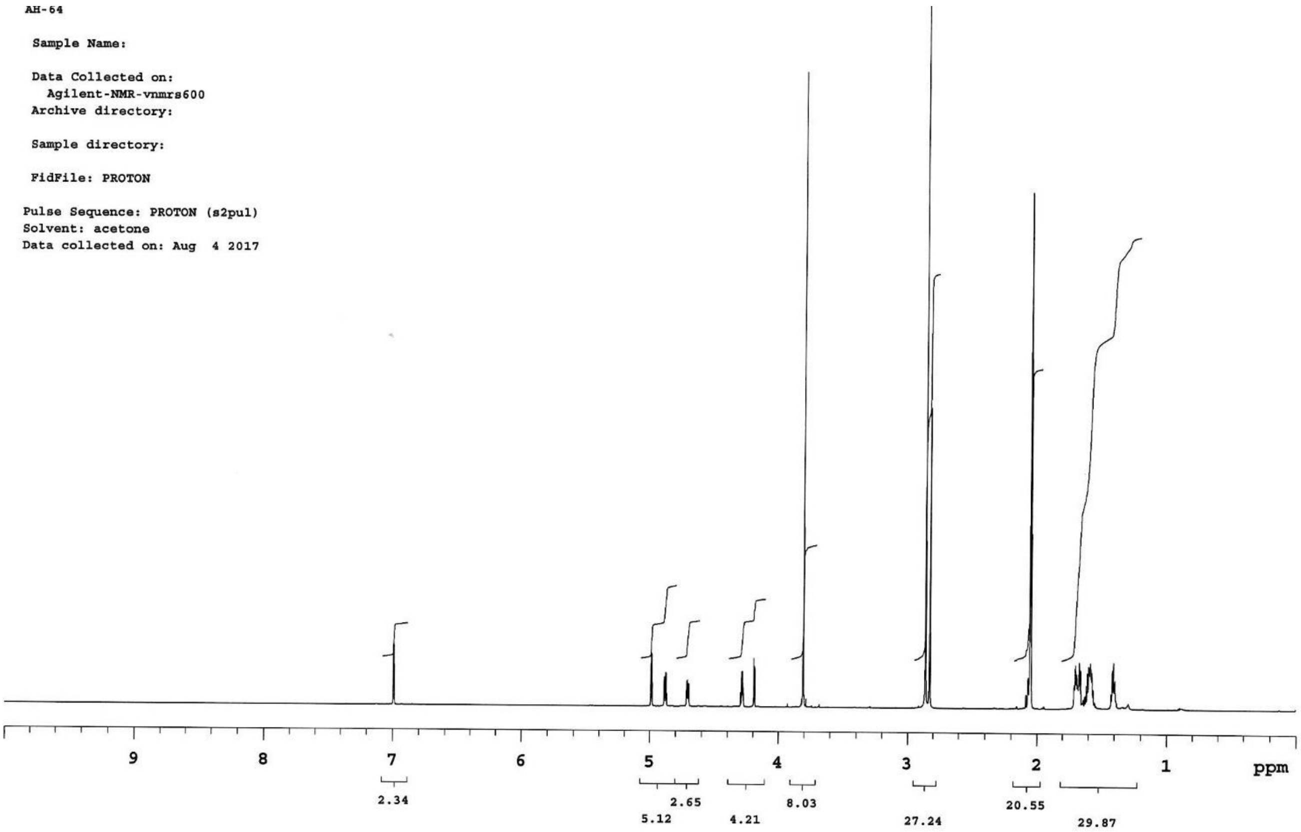

AH-64

INDEX	FREQUENCY	PPM	HEIGHT	INDEX	FREQUENCY	PPM	HEIGHT
1	4196.2	6.995	64.2	51	983.0	1.639	10.2
2	4194.7	6.992	44.9	52	977.1	1.629	13.7
3	4192.9	6.989	64.0	53	970.4	1.618	25.3
4	4191.5	6.987	43.0	54	964.2	1.607	38.5
5	2993.4	4.990	57.7	55	962.2	1.604	20.1
6	2989.0	4.982	55.8	56	958.4	1.598	45.4
7	2932.3	4.888	31.4	57	956.0	1.594	41.1
8	2929.4	4.883	31.5	58	952.2	1.587	48.7
9	2925.3	4.876	36.3	59	950.2	1.584	48.6
10	2922.1	4.871	35.4	60	946.9	1.578	34.2
11	2829.3	4.716	27.2	61	943.7	1.573	39.1
12	2828.5	4.715	21.1	62	940.2	1.567	15.6
13	2825.8	4.710	28.4	63	937.8	1.563	21.1
14	2824.6	4.709	21.7	64	930.5	1.551	7.4
15	2822.3	4.705	24.6	65	855.4	1.426	16.5
16	2818.5	4.698	24.7	66	849.5	1.416	41.6
17	2817.6	4.697	18.3	67	843.6	1.406	49.7
18	2578.1	4.298	15.6	68	837.8	1.397	30.7
19	2574.0	4.291	37.8	69	832.2	1.387	9.5
20	2572.0	4.287	19.8	70	-0.0	-0.000	41.3
21	2570.2	4.284	38.3				
22	2567.9	4.281	25.4				
23	2566.1	4.278	20.6				
24	2564.1	4.274	14.6				
25	2515.9	4.194	52.0				
26	2511.8	4.187	43.1				
27	2291.8	3.820	9.2				
28	2283.5	3.807	671.5				
29	2279.1	3.799	13.6				
30	2277.4	3.796	8.4				
31	2270.3	3.785	9.0				
32	1715.7	2.860	1134.6				
33	1696.7	2.828	268.6				
34	1695.8	2.827	317.7				
35	1254.4	2.091	12.5				
36	1253.6	2.090	15.1				
37	1245.1	2.075	31.6				
38	1242.7	2.072	33.5				
39	1240.7	2.068	33.3				
40	1237.1	2.062	193.8				
41	1235.1	2.059	376.5				
42	1232.7	2.055	545.2				
43	1230.7	2.051	369.8				
44	1228.3	2.048	184.8				
45	1026.2	1.711	26.1				
46	1020.3	1.701	46.7				
47	1014.4	1.691	37.1				
48	1010.3	1.684	25.9				
49	1002.4	1.671	49.7				
50	996.8	1.662	41.8				

Figure S13. ^{13}C-NMR spectrum of (+)-**10**$_{Br}$ in acetone-d$_6$ (150 MHz)

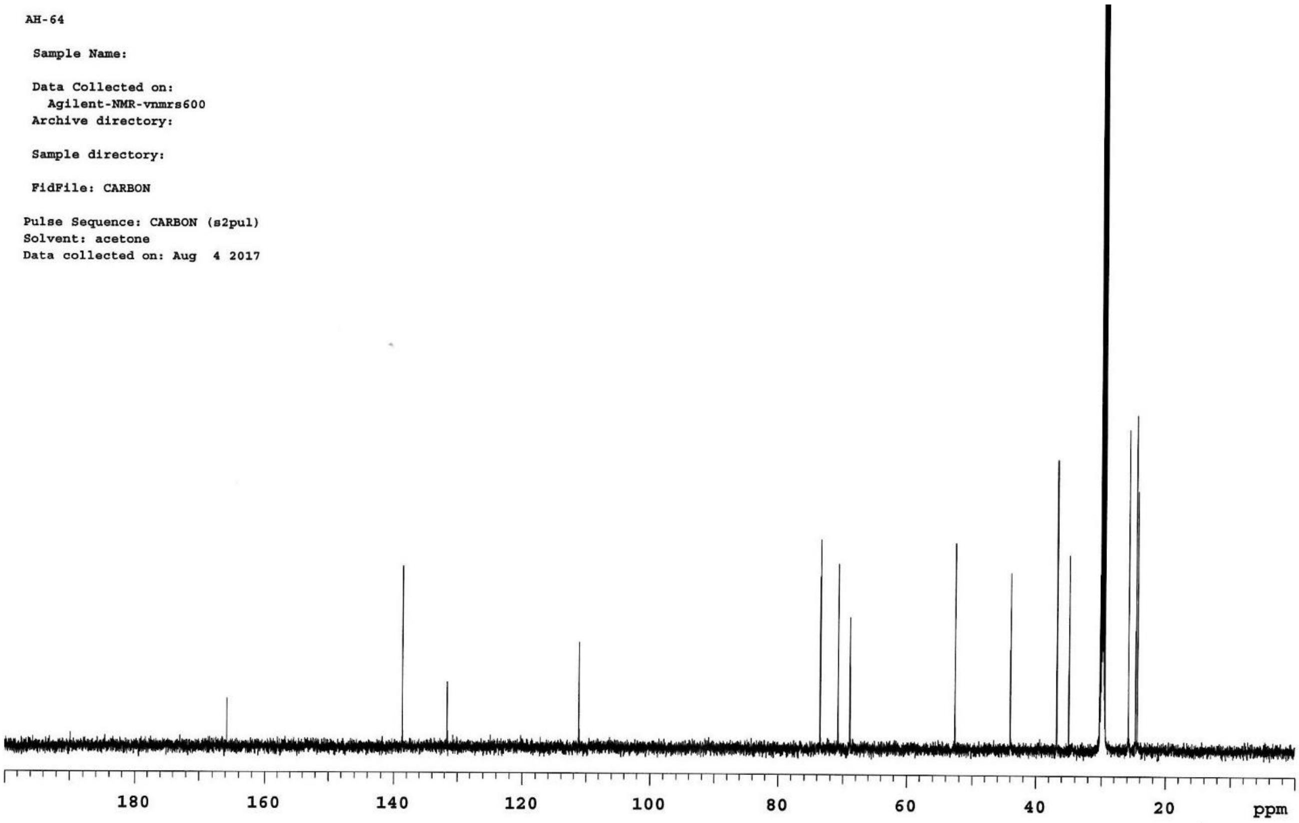

INDEX	FREQUENCY	PPM	HEIGHT
1	25005.9	165.773	10.1
2	20916.1	138.660	37.6
3	19848.0	131.579	13.6
4	16762.7	111.126	21.9
5	11096.1	73.560	43.4
6	10678.8	70.794	38.4
7	10403.7	68.970	27.3
8	10392.2	68.893	17.2
9	7936.9	52.616	42.9
10	6638.7	44.010	36.8
11	5548.6	36.784	60.1
12	5268.9	34.929	25.5
13	5266.6	34.914	40.5
14	4577.6	30.347	11.4
15	4559.1	30.224	157.8
16	4540.6	30.101	595.7
17	4521.0	29.971	1274.4
18	4501.3	29.841	1321.1
19	4482.8	29.718	952.9
20	4463.2	29.588	606.2
21	4443.5	29.458	213.9
22	3896.8	25.833	66.4
23	3726.8	24.706	69.3
24	3684.1	24.423	53.7

Figure S14. ^{1}H-NMR spectrum of (-)-**1**$_{Br}$ in methanol-d$_4$ (600 MHz)

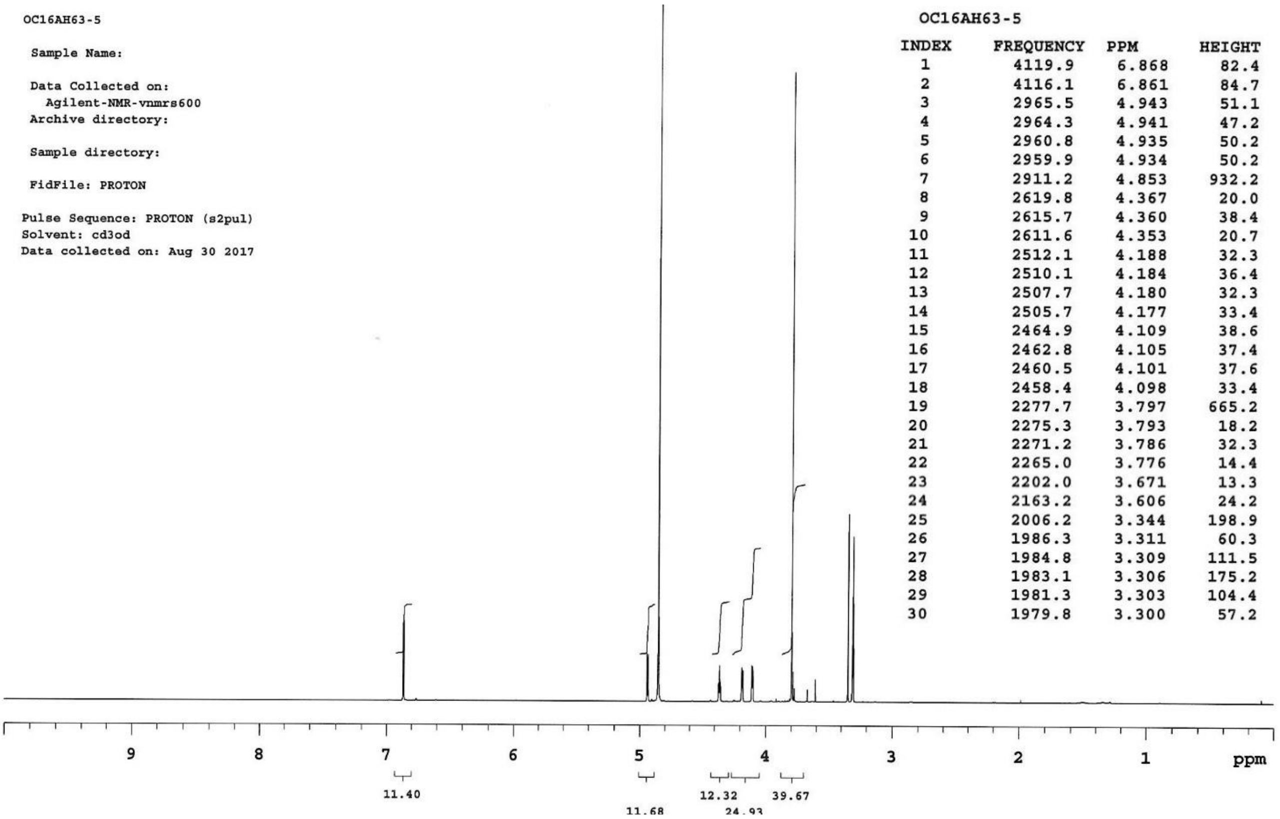

OC16AH63-5

INDEX	FREQUENCY	PPM	HEIGHT
1	4119.9	6.868	82.4
2	4116.1	6.861	84.7
3	2965.5	4.943	51.1
4	2964.3	4.941	47.2
5	2960.8	4.935	50.2
6	2959.9	4.934	50.2
7	2911.2	4.853	932.2
8	2619.8	4.367	20.0
9	2615.7	4.360	38.4
10	2611.6	4.353	20.7
11	2512.1	4.188	32.3
12	2510.1	4.184	36.4
13	2507.7	4.180	32.3
14	2505.7	4.177	33.4
15	2464.9	4.109	38.6
16	2462.8	4.105	37.4
17	2460.5	4.101	37.6
18	2458.4	4.098	33.4
19	2277.7	3.797	665.2
20	2275.3	3.793	18.2
21	2271.2	3.786	32.3
22	2265.0	3.776	14.4
23	2202.0	3.671	13.3
24	2163.2	3.606	24.2
25	2006.2	3.344	198.9
26	1986.3	3.311	60.3
27	1984.8	3.309	111.5
28	1983.1	3.306	175.2
29	1981.3	3.303	104.4
30	1979.8	3.300	57.2

Figure S15. ^{13}C-NMR spectrum of (-)-**1**$_{Br}$ in methanol-d$_4$ (150 MHz)

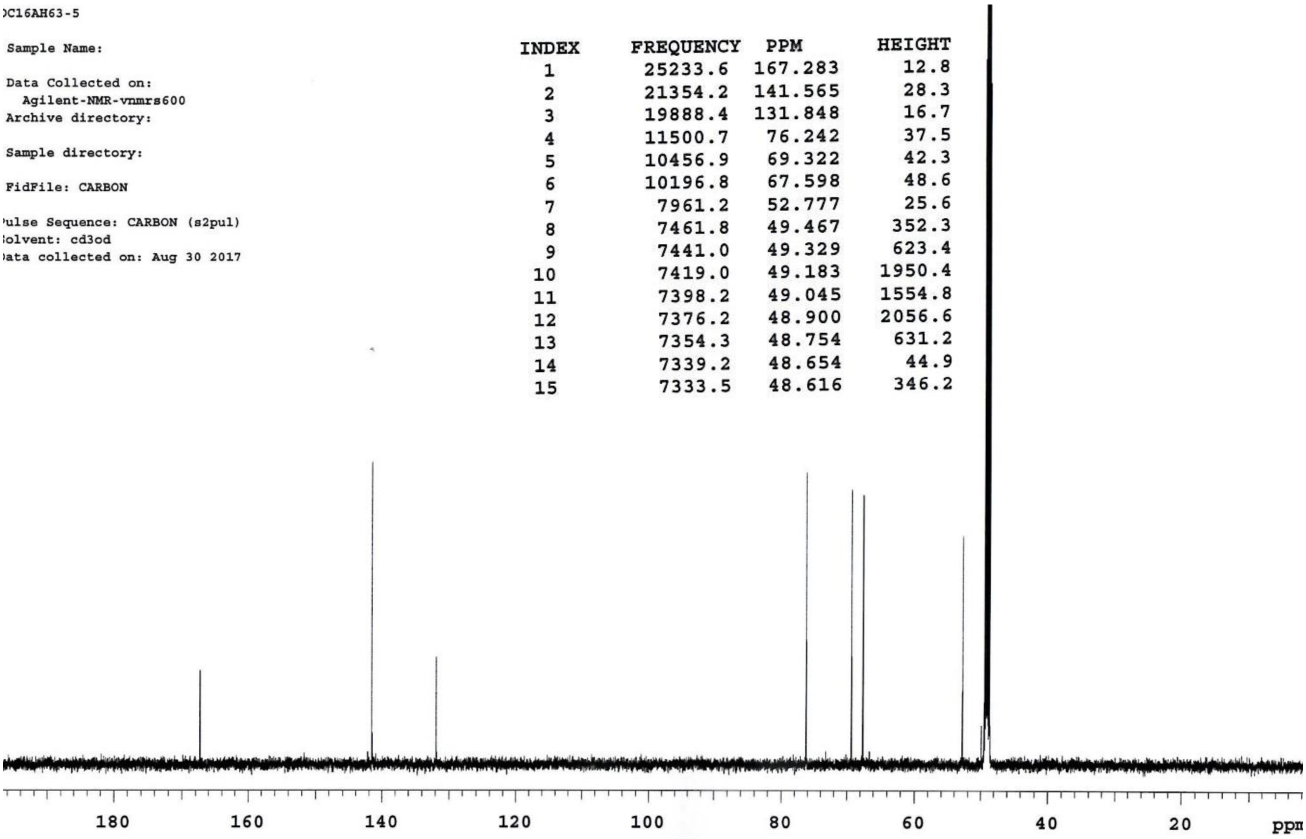

INDEX	FREQUENCY	PPM	HEIGHT
1	25233.6	167.283	12.8
2	21354.2	141.565	28.3
3	19888.4	131.848	16.7
4	11500.7	76.242	37.5
5	10456.9	69.322	42.3
6	10196.8	67.598	48.6
7	7961.2	52.777	25.6
8	7461.8	49.467	352.3
9	7441.0	49.329	623.4
10	7419.0	49.183	1950.4
11	7398.2	49.045	1554.8
12	7376.2	48.900	2056.6
13	7354.3	48.754	631.2
14	7339.2	48.654	44.9
15	7333.5	48.616	346.2

Figure S16. ^{1}H-NMR spectrum of (+)-**1**Br in methanol-d4 (600 MHz)

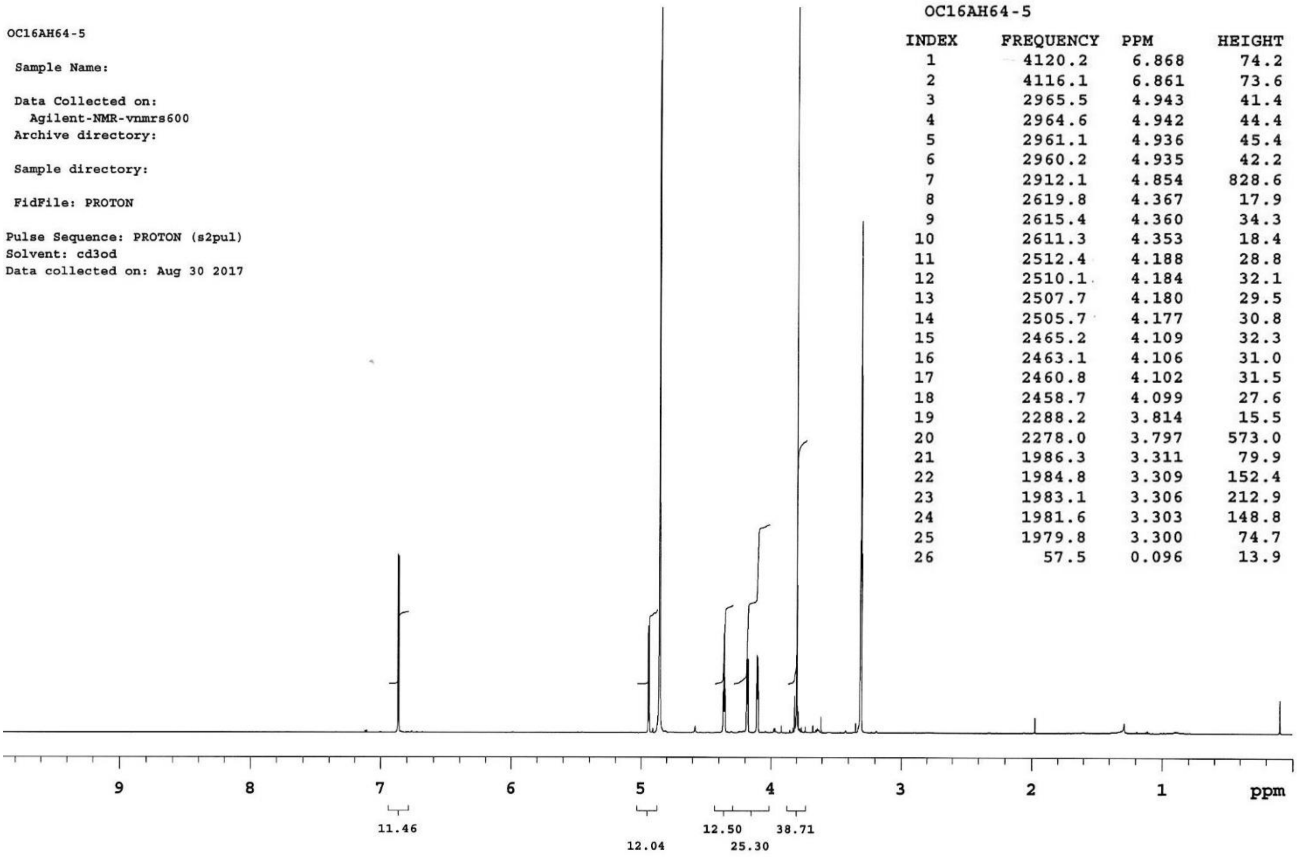

INDEX	FREQUENCY	PPM	HEIGHT
1	4120.2	6.868	74.2
2	4116.1	6.861	73.6
3	2965.5	4.943	41.4
4	2964.6	4.942	44.4
5	2961.1	4.936	45.4
6	2960.2	4.935	42.2
7	2912.1	4.854	828.6
8	2619.8	4.367	17.9
9	2615.4	4.360	34.3
10	2611.3	4.353	18.4
11	2512.4	4.188	28.8
12	2510.1	4.184	32.1
13	2507.7	4.180	29.5
14	2505.7	4.177	30.8
15	2465.2	4.109	32.3
16	2463.1	4.106	31.0
17	2460.8	4.102	31.5
18	2458.7	4.099	27.6
19	2288.2	3.814	15.5
20	2278.0	3.797	573.0
21	1986.3	3.311	79.9
22	1984.8	3.309	152.4
23	1983.1	3.306	212.9
24	1981.6	3.303	148.8
25	1979.8	3.300	74.7
26	57.5	0.096	13.9

Figure S17. ^{13}C-NMR spectrum of (+)-**1**Br in methanol-d4 (150 MHz)

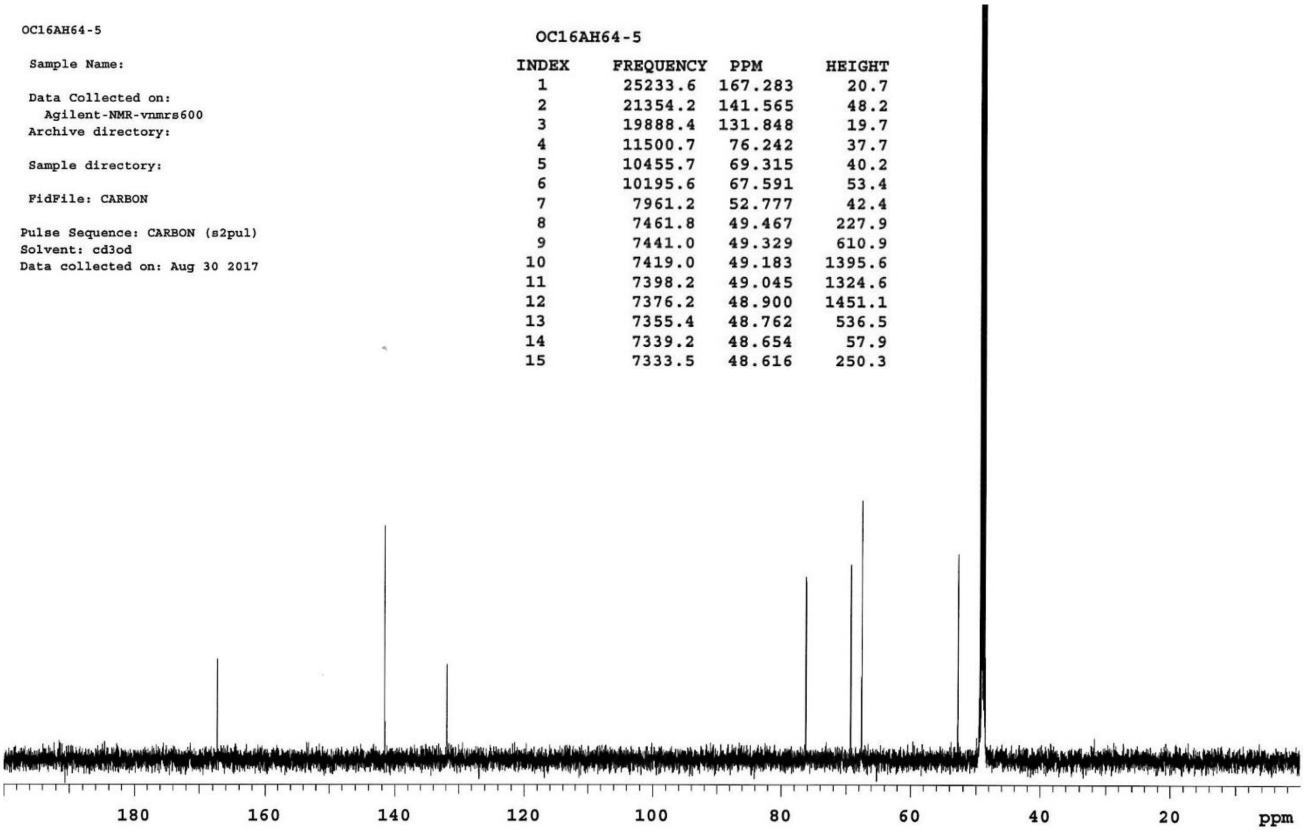

INDEX	FREQUENCY	PPM	HEIGHT
1	25233.6	167.283	20.7
2	21354.2	141.565	48.2
3	19888.4	131.848	19.7
4	11500.7	76.242	37.7
5	10455.7	69.315	40.2
6	10195.6	67.591	53.4
7	7961.2	52.777	42.4
8	7461.8	49.467	227.9
9	7441.0	49.329	610.9
10	7419.0	49.183	1395.6
11	7398.2	49.045	1324.6
12	7376.2	48.900	1451.1
13	7355.4	48.762	536.5
14	7339.2	48.654	57.9
15	7333.5	48.616	250.3

Figure S18. ^{1}H-NMR spectrum of (-)-**10**ɪ in $CDCl_3$ (600 MHz)

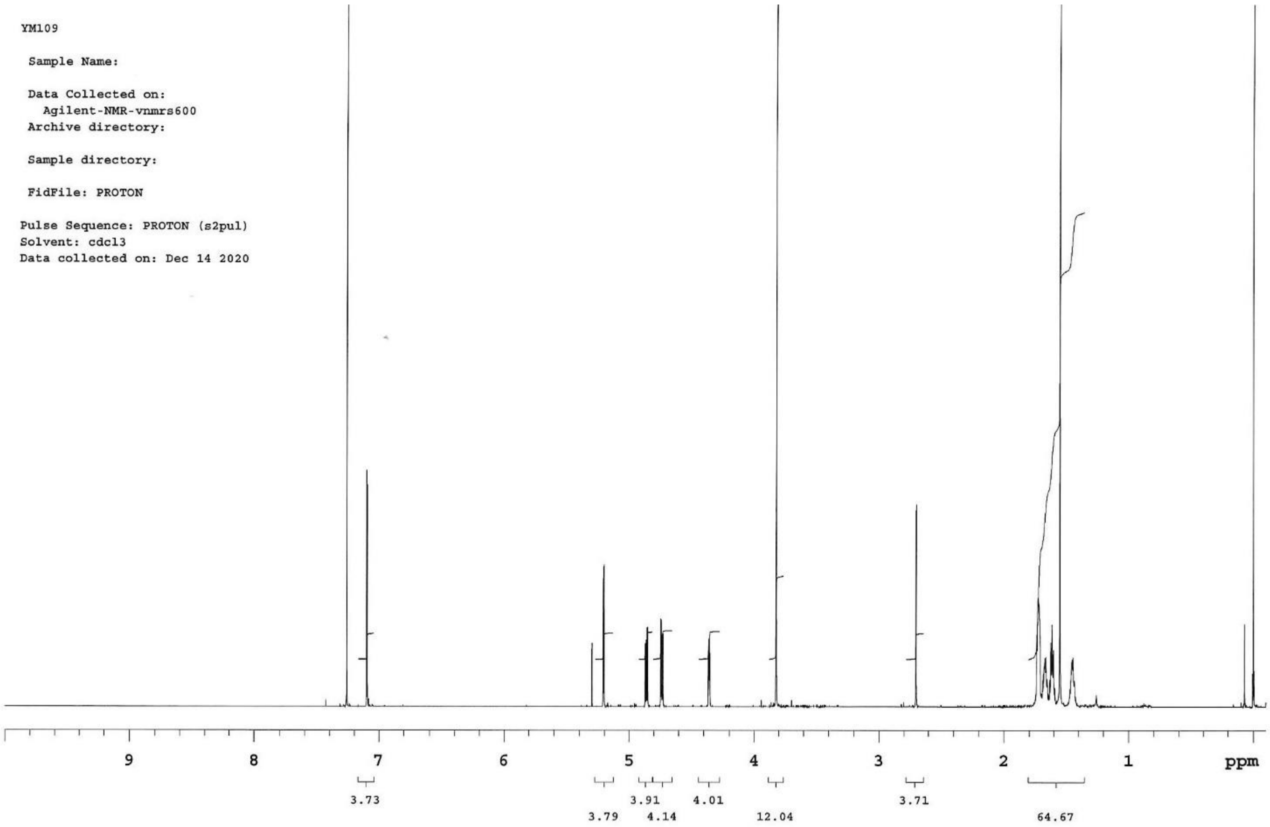

YM109

INDEX	FREQUENCY	PPM	HEIGHT	INDEX	FREQUENCY	PPM	HEIGHT
1	4355.8	7.261	531.7	51	854.5	1.424	4.1
2	4259.5	7.100	93.2	52	753.0	1.255	4.7
3	4256.3	7.095	87.6	53	41.4	0.069	32.8
4	3179.7	5.300	25.1	54	3.2	0.005	13.3
5	3125.7	5.210	55.3	55	0.0	0.000	536.4
6	3122.5	5.205	56.1	56	-3.2	-0.005	14.5
7	2921.2	4.869	25.1				
8	2917.1	4.863	26.4				
9	2913.3	4.856	31.2				
10	2909.1	4.849	31.5				
11	2846.1	4.744	34.8				
12	2843.1	4.739	34.2				
13	2838.4	4.732	28.5				
14	2835.2	4.726	29.0				
15	2620.4	4.368	20.8				
16	2618.1	4.364	23.9				
17	2616.9	4.362	24.7				
18	2616.3	4.361	26.4				
19	2614.8	4.359	26.7				
20	2613.9	4.357	24.6				
21	2613.1	4.356	23.2				
22	2610.7	4.352	21.2				
23	2293.5	3.823	593.5				
24	1623.0	2.705	77.5				
25	1620.7	2.702	80.0				
26	1042.3	1.737	23.5				
27	1038.2	1.731	43.2				
28	1035.3	1.726	43.4				
29	1032.3	1.721	40.1				
30	1030.0	1.717	34.3				
31	1022.9	1.705	5.3				
32	1018.5	1.698	5.2				
33	1014.4	1.691	10.6				
34	1011.5	1.686	13.2				
35	1008.3	1.681	16.0				
36	1003.3	1.672	19.3				
37	999.7	1.667	19.2				
38	993.3	1.656	11.0				
39	987.1	1.645	5.4				
40	980.4	1.634	10.8				
41	974.5	1.624	25.4				
42	968.6	1.615	32.5				
43	962.5	1.604	22.4				
44	956.9	1.595	7.6				
45	930.8	1.552	833.3				
46	881.8	1.470	6.3				
47	875.6	1.460	13.6				
48	870.9	1.452	18.4				
49	866.8	1.445	19.6				
50	860.9	1.435	10.4				

Figure S19. ^{13}C-NMR spectrum of (-)-**10**$_{l}$ in $CDCl_3$ (150 MHz)

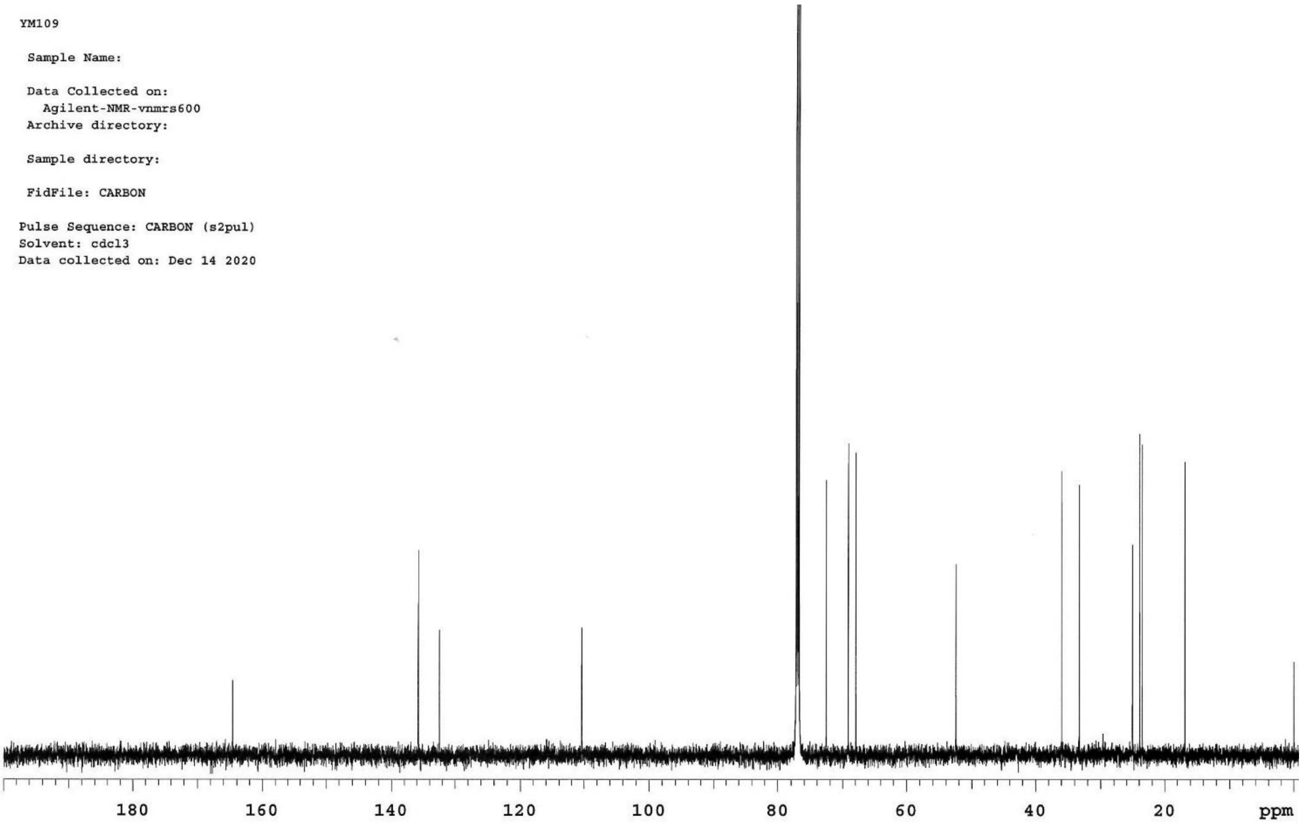

YM109

INDEX	FREQUENCY	PPM	HEIGHT
1	24838.3	164.662	15.3
2	20486.1	135.810	42.0
3	19986.7	132.499	25.7
4	16652.9	110.398	26.1
5	11649.9	77.231	1316.3
6	11617.5	77.017	1199.5
7	11586.3	76.810	1306.7
8	10949.3	72.587	56.5
9	10431.5	69.154	64.1
10	10250.0	67.951	62.1
11	7914.9	52.471	39.3
12	5428.4	35.987	58.4
13	5020.4	33.282	55.6
14	3784.6	25.090	43.2
15	3613.6	23.956	66.1
16	3548.8	23.526	63.9
17	2539.7	16.836	60.4
18	0.0	0.000	19.1

Figure S20. ^{1}H-NMR spectrum of (+)-**10**I in $CDCl_3$ (400 MHz)

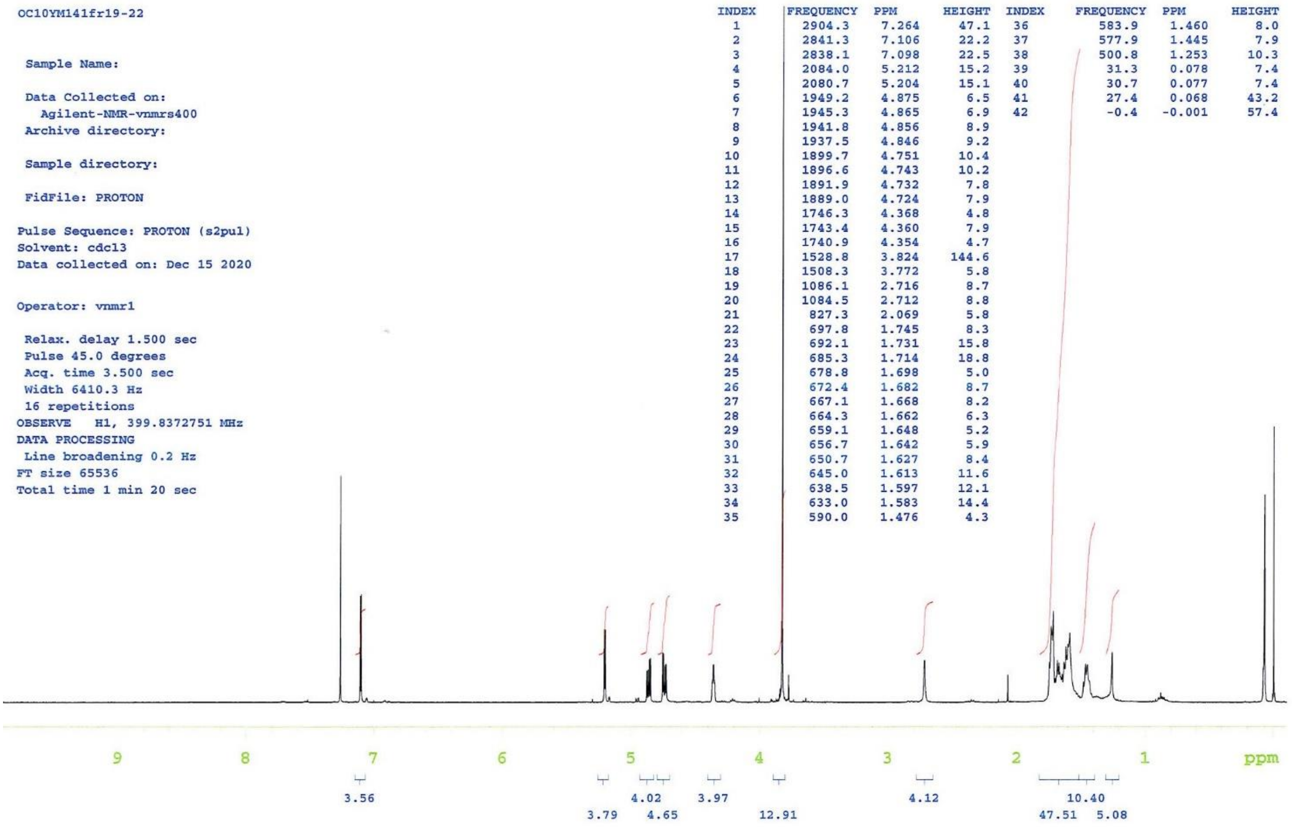

Figure S21. ^{13}C-NMR spectrum of (+)-**10**I in $CDCl_3$ (100 MHz)

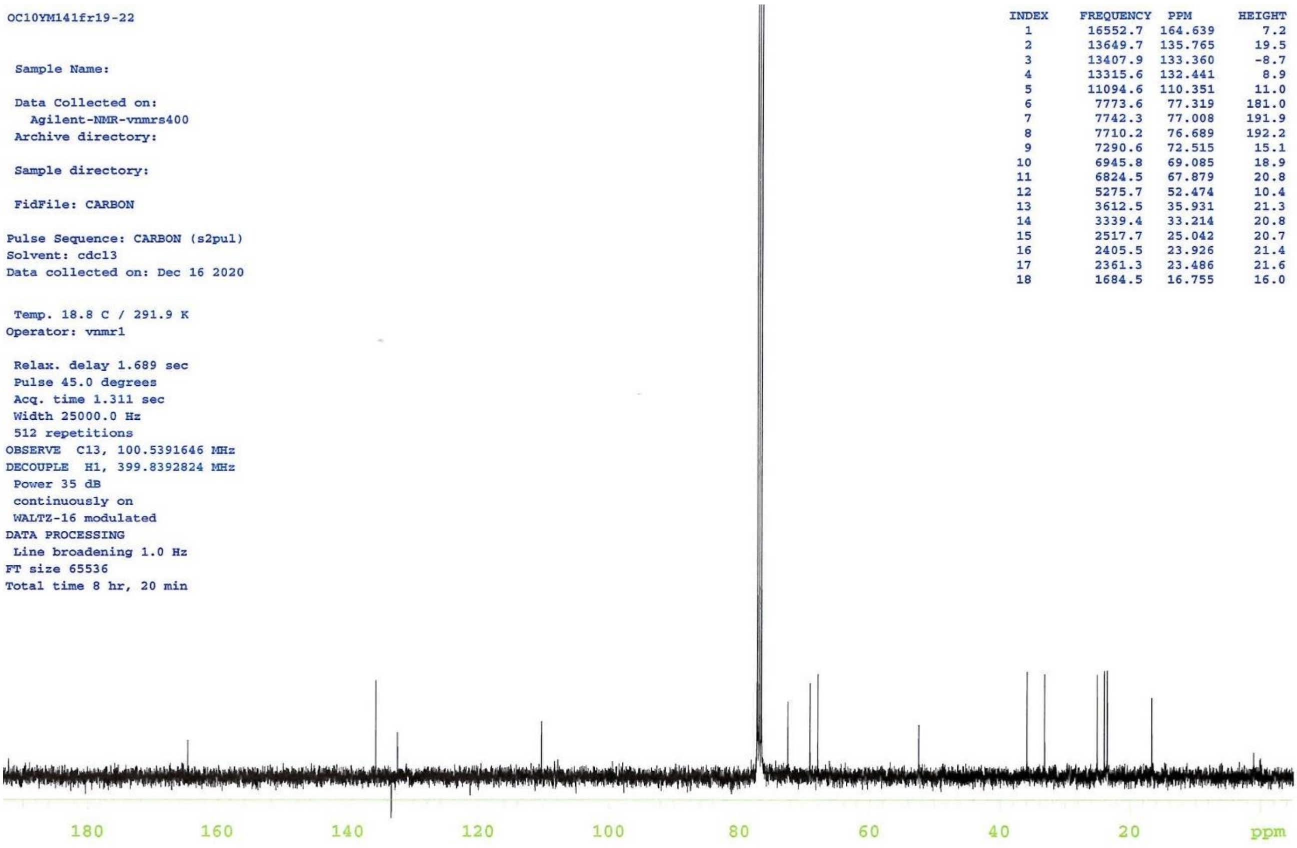

Figure S22. ^{1}H-NMR spectrum of (-)-**1**ı in acetone-d$_6$ (600 MHz)

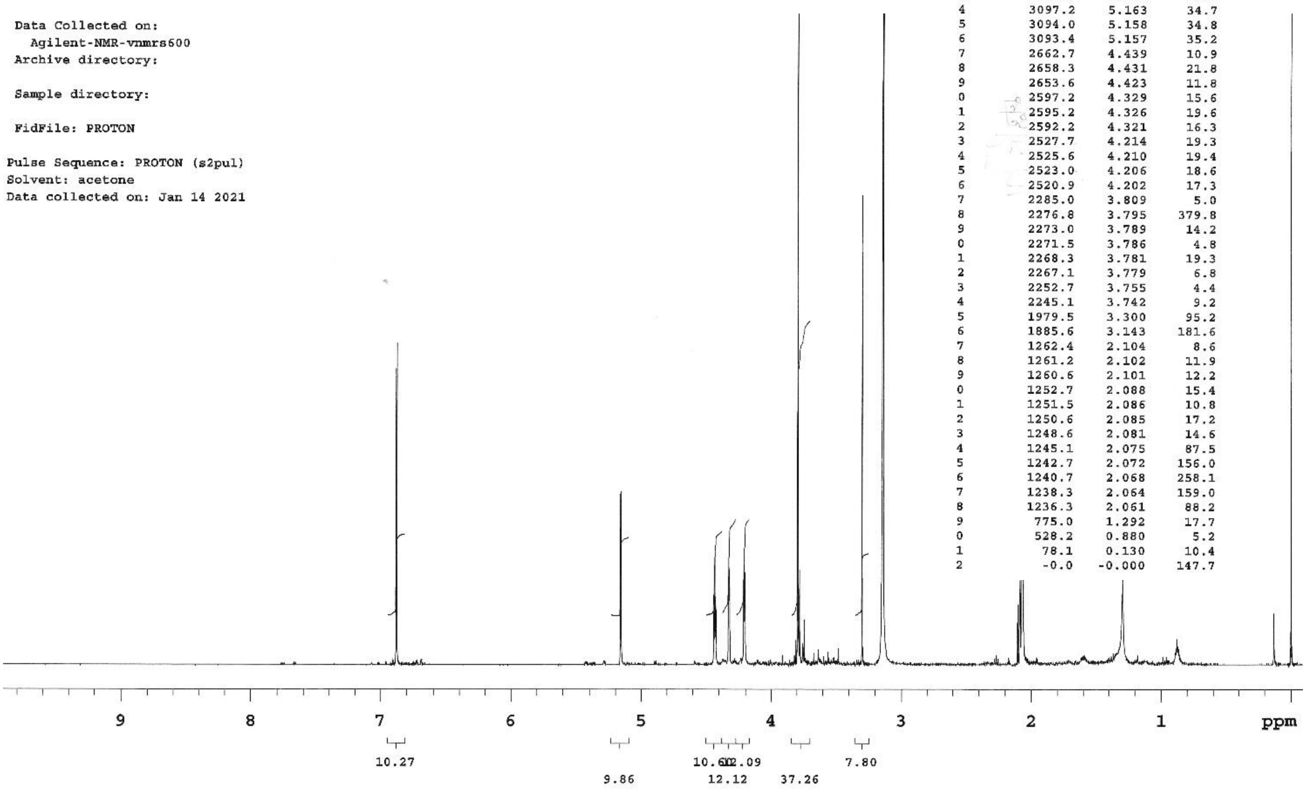

IDEX	FREQUENCY	PPM	HEIGHT
1	4128.1	6.881	60.0
2	4123.7	6.874	65.1
3	3097.8	5.164	33.7
4	3097.2	5.163	34.7
5	3094.0	5.158	34.8
6	3093.4	5.157	35.2
7	2662.7	4.439	10.9
8	2658.3	4.431	21.8
9	2653.6	4.423	11.8
0	2597.2	4.329	15.6
1	2595.2	4.326	19.6
2	2592.2	4.321	16.3
3	2527.7	4.214	19.3
4	2525.6	4.210	19.4
5	2523.0	4.206	18.6
6	2520.9	4.202	17.3
7	2285.0	3.809	5.0
8	2276.8	3.795	379.8
9	2273.0	3.789	14.2
0	2271.5	3.786	4.8
1	2268.3	3.781	19.3
2	2267.1	3.779	6.8
3	2252.7	3.755	4.4
4	2245.1	3.742	9.2
5	1979.5	3.300	95.2
6	1885.6	3.143	181.6
7	1262.4	2.104	8.6
8	1261.2	2.102	11.9
9	1260.6	2.101	12.2
0	1252.7	2.088	15.4
1	1251.5	2.086	10.8
2	1250.6	2.085	17.2
3	1248.6	2.081	14.6
4	1245.1	2.075	87.5
5	1242.7	2.072	156.0
6	1240.7	2.068	258.1
7	1238.3	2.064	159.0
8	1236.3	2.061	88.2
9	775.0	1.292	17.7
0	528.2	0.880	5.2
1	78.1	0.130	10.4
2	-0.0	-0.000	147.7

Figure S23. ^{13}C-NMR spectrum of (-)-**1**ı in acetone-d$_6$ (150 MHz)

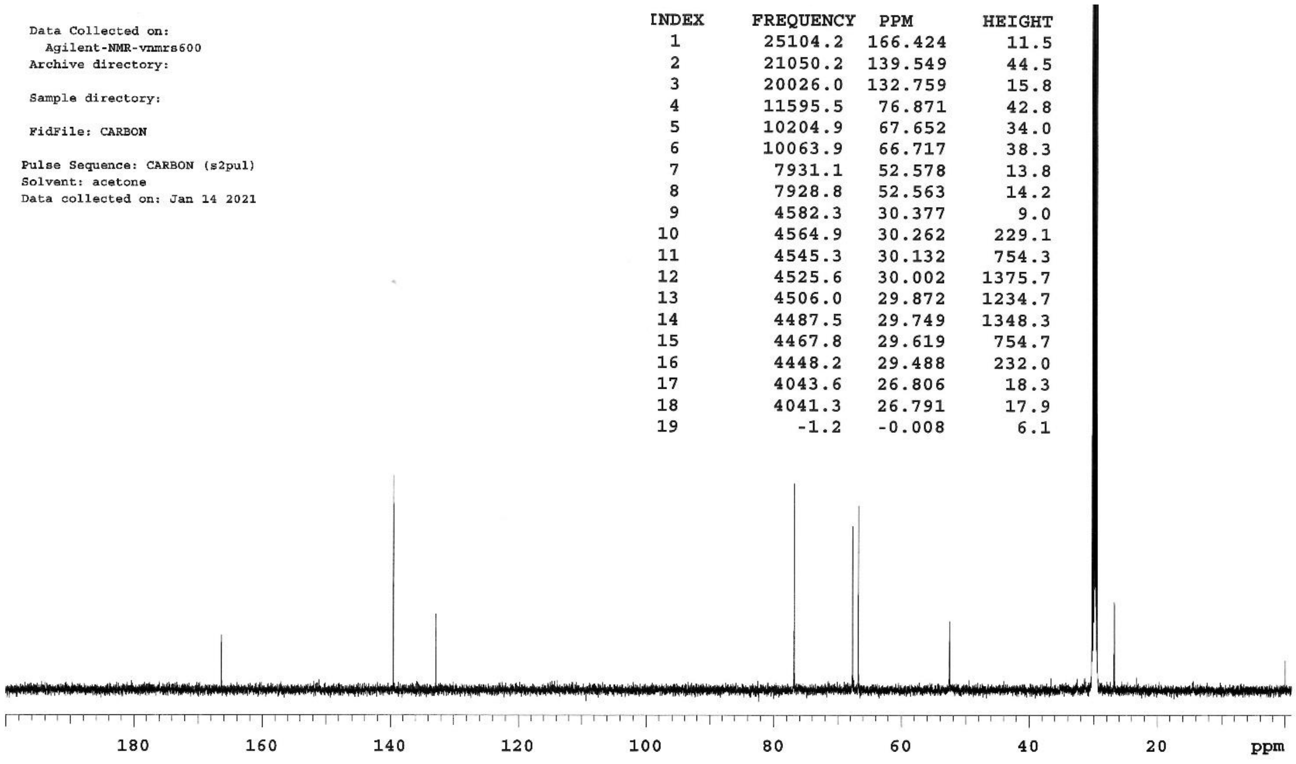

INDEX	FREQUENCY	PPM	HEIGHT
1	25104.2	166.424	11.5
2	21050.2	139.549	44.5
3	20026.0	132.759	15.8
4	11595.5	76.871	42.8
5	10204.9	67.652	34.0
6	10063.9	66.717	38.3
7	7931.1	52.578	13.8
8	7928.8	52.563	14.2
9	4582.3	30.377	9.0
10	4564.9	30.262	229.1
11	4545.3	30.132	754.3
12	4525.6	30.002	1375.7
13	4506.0	29.872	1234.7
14	4487.5	29.749	1348.3
15	4467.8	29.619	754.7
16	4448.2	29.488	232.0
17	4043.6	26.806	18.3
18	4041.3	26.791	17.9
19	-1.2	-0.008	6.1

Figure S24. ^{1}H-NMR spectrum of (+)-**1**ɪ in acetone-d$_6$ (600 MHz)

Data Collected on:
Agilent-NMR-vnmrs600
Archive directory:

Sample directory:

FidFile: PROTON

Pulse Sequence: PROTON (s2pul)
Solvent: acetone
Data collected on: Jan 8 2021

INDEX	FREQUENCY	PPM	HEIGHT	INDEX	FREQUENCY	PPM	HEIGHT
1	4128.4	6.882	49.3	51	528.5	0.881	5.7
2	4124.0	6.874	43.9	52	78.3	0.131	8.2
3	3097.8	5.164	20.5	53	3.5	0.006	9.3
4	3097.2	5.163	23.5	54	0.0	0.000	278.8
5	3094.3	5.158	23.0	55	-3.2	-0.005	9.0
6	3093.7	5.157	24.1				
7	2661.8	4.437	7.3				
8	2657.1	4.429	14.6				
9	2652.4	4.421	7.6				
10	2597.5	4.330	9.1				
11	2596.9	4.329	9.8				
12	2595.5	4.326	10.9				
13	2594.9	4.326	11.2				
14	2594.0	4.324	11.0				
15	2593.4	4.323	10.1				
16	2591.9	4.321	10.0				
17	2527.1	4.213	16.8				
18	2525.0	4.209	16.3				
19	2522.4	4.205	15.4				
20	2520.3	4.201	15.3				
21	2367.2	3.946	11.7				
22	2276.8	3.795	321.8				
23	2274.7	3.792	5.4				
24	2273.0	3.789	4.9				
25	2268.0	3.781	11.2				
26	2267.1	3.779	4.0				
27	2264.5	3.775	4.6				
28	2238.1	3.731	9.8				
29	2235.4	3.726	4.3				
30	2169.4	3.616	9.7				
31	2135.1	3.559	9.1				
32	1979.2	3.299	12.7				
33	1861.6	3.103	281.9				
34	1261.5	2.103	11.8				
35	1260.3	2.101	17.4				
36	1259.7	2.100	17.3				
37	1253.0	2.089	16.2				
38	1252.1	2.087	21.9				
39	1250.6	2.085	16.4				
40	1249.8	2.083	23.8				
41	1248.6	2.081	15.0				
42	1247.7	2.080	20.8				
43	1244.2	2.074	164.6				
44	1242.1	2.071	278.2				
45	1239.8	2.067	492.8				
46	1237.7	2.063	272.2				
47	1235.4	2.059	164.3				
48	1211.9	2.020	4.3				
49	775.6	1.293	20.6				
50	531.1	0.885	3.9				

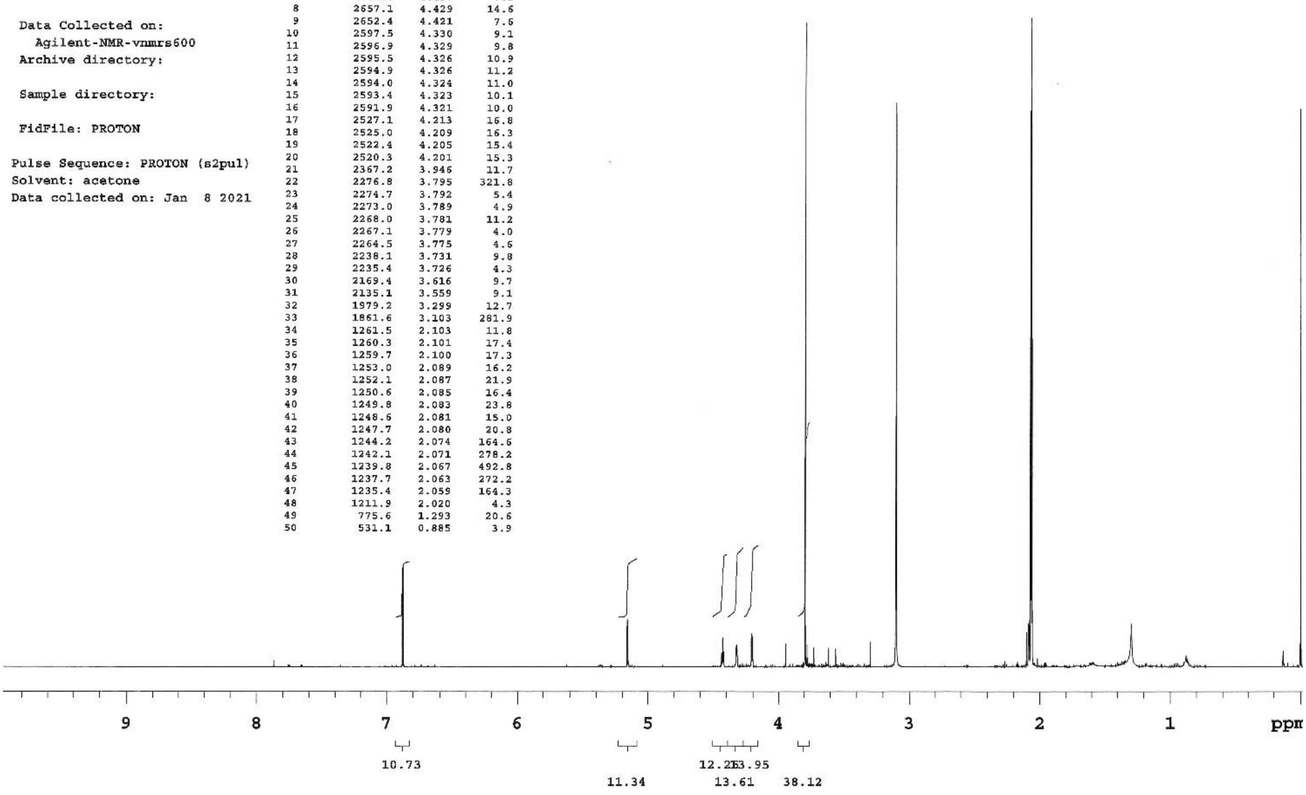

Figure S25. ^{13}C-NMR spectrum of (+)-**1**ɪ in acetone-d$_6$ (150 MHz)

Data Collected on:
Agilent-NMR-vnmrs600
Archive directory:

Sample directory:

FidFile: CARBON

Pulse Sequence: CARBON (s2pul)
Solvent: acetone
Data collected on: Jan 8 2021

INDEX	FREQUENCY	PPM	HEIGHT
1	25104.2	166.424	13.1
2	21050.2	139.549	54.4
3	20028.3	132.775	13.8
4	11599.0	76.894	46.4
5	10204.9	67.652	44.8
6	10066.2	66.732	50.9
7	7929.9	52.570	30.7
8	4582.3	30.377	28.0
9	4563.8	30.255	583.1
10	4545.3	30.132	1544.1
11	4525.6	30.002	3866.7
12	4506.0	29.872	4726.9
13	4486.3	29.741	3477.2
14	4467.8	29.619	1526.3
15	4448.2	29.488	645.7
16	4042.4	26.799	48.9
17	-0.0	-0.000	26.2

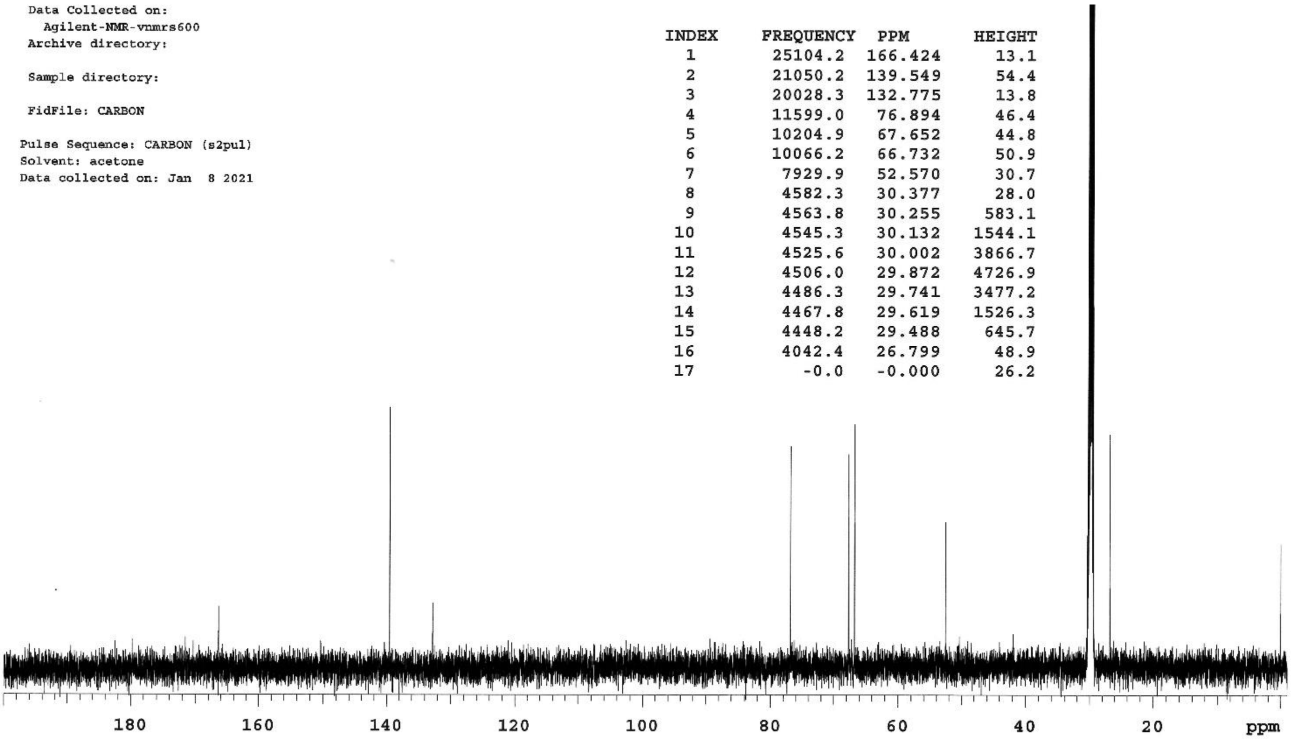

Figure S26. ^{1}H-NMR spectrum of (-)-**11** in methanol-d4 (600 MHz)

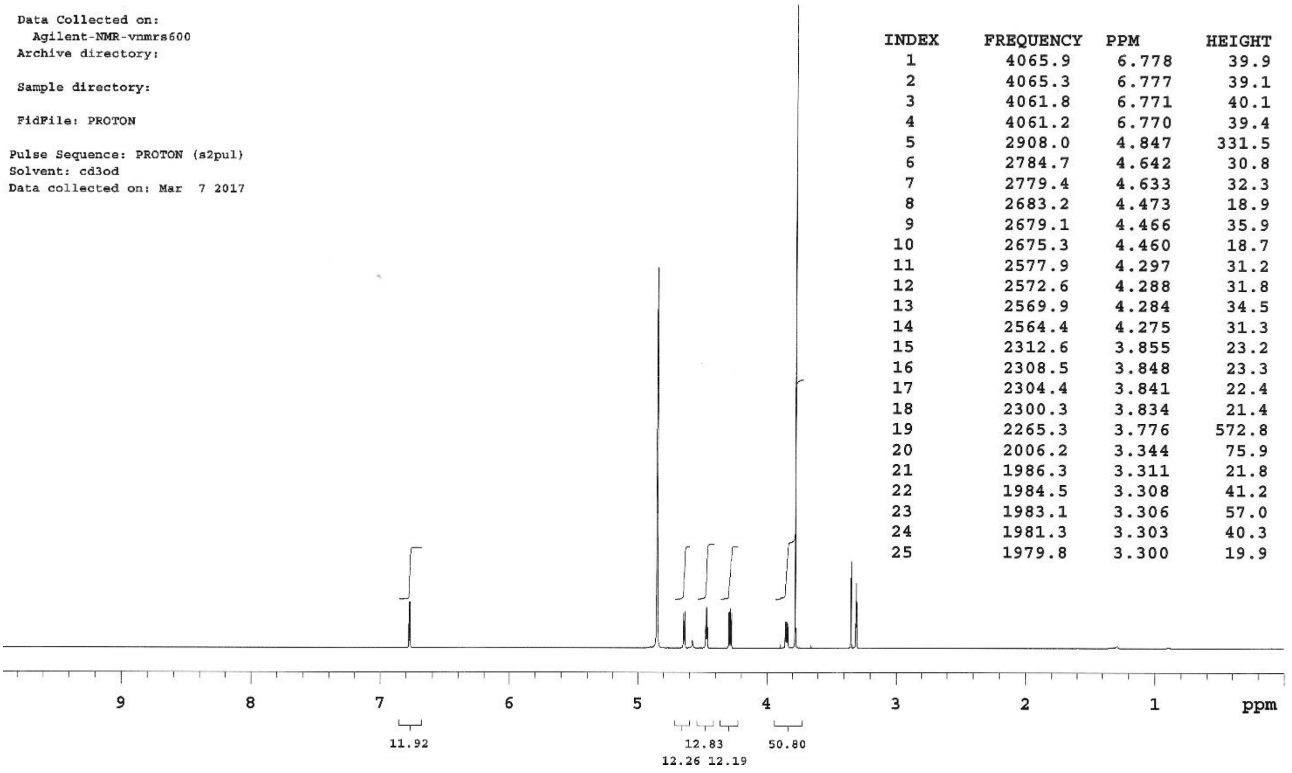

INDEX	FREQUENCY	PPM	HEIGHT
1	4065.9	6.778	39.9
2	4065.3	6.777	39.1
3	4061.8	6.771	40.1
4	4061.2	6.770	39.4
5	2908.0	4.847	331.5
6	2784.7	4.642	30.8
7	2779.4	4.633	32.3
8	2683.2	4.473	18.9
9	2679.1	4.466	35.9
10	2675.3	4.460	18.7
11	2577.9	4.297	31.2
12	2572.6	4.288	31.8
13	2569.9	4.284	34.5
14	2564.4	4.275	31.3
15	2312.6	3.855	23.2
16	2308.5	3.848	23.3
17	2304.4	3.841	22.4
18	2300.3	3.834	21.4
19	2265.3	3.776	572.8
20	2006.2	3.344	75.9
21	1986.3	3.311	21.8
22	1984.5	3.308	41.2
23	1983.1	3.306	57.0
24	1981.3	3.303	40.3
25	1979.8	3.300	19.9

Figure S27. ^{13}C-NMR spectrum of (-)-**11** in methanol-d4 (150 MHz)

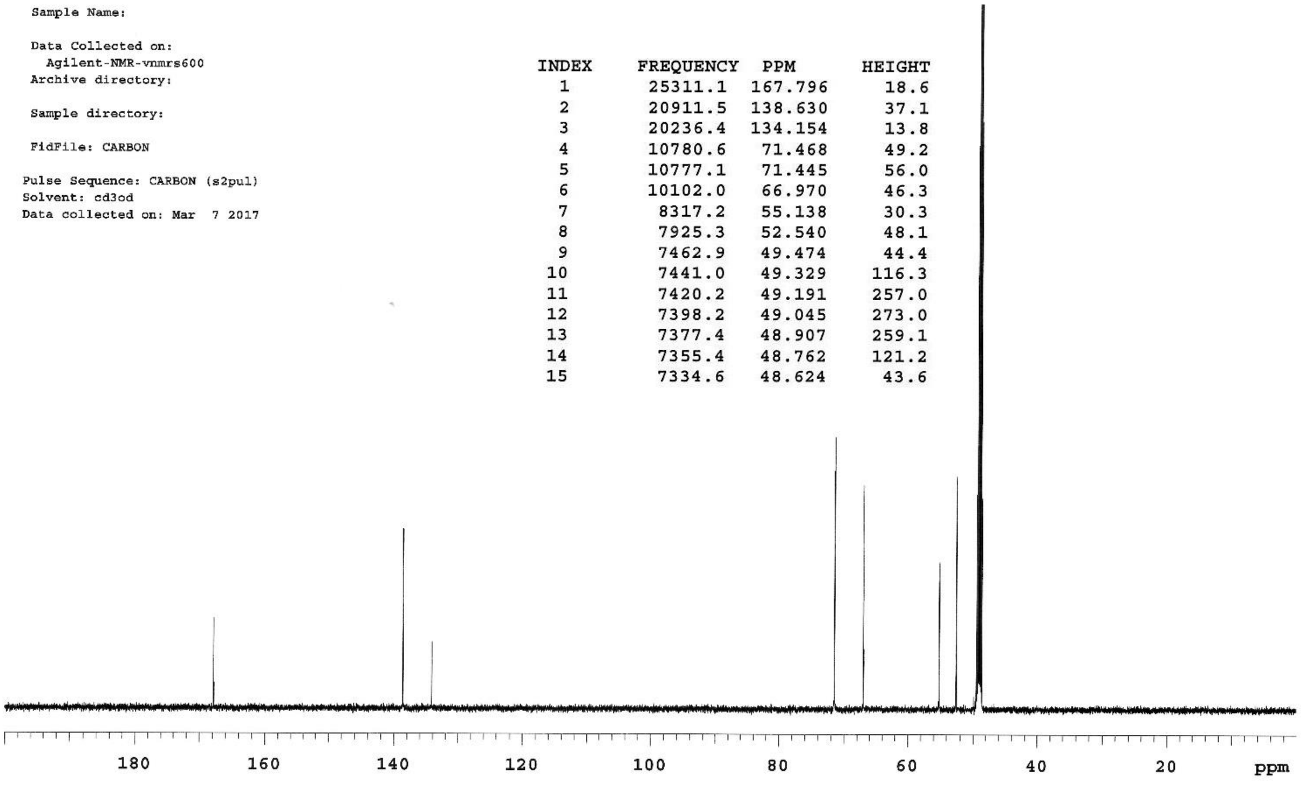

INDEX	FREQUENCY	PPM	HEIGHT
1	25311.1	167.796	18.6
2	20911.5	138.630	37.1
3	20236.4	134.154	13.8
4	10780.6	71.468	49.2
5	10777.1	71.445	56.0
6	10102.0	66.970	46.3
7	8317.2	55.138	30.3
8	7925.3	52.540	48.1
9	7462.9	49.474	44.4
10	7441.0	49.329	116.3
11	7420.2	49.191	257.0
12	7398.2	49.045	273.0
13	7377.4	48.907	259.1
14	7355.4	48.762	121.2
15	7334.6	48.624	43.6

Figure S28. ^{1}H-NMR spectrum of (-)-**12** in methanol-d4 (600 MHz)

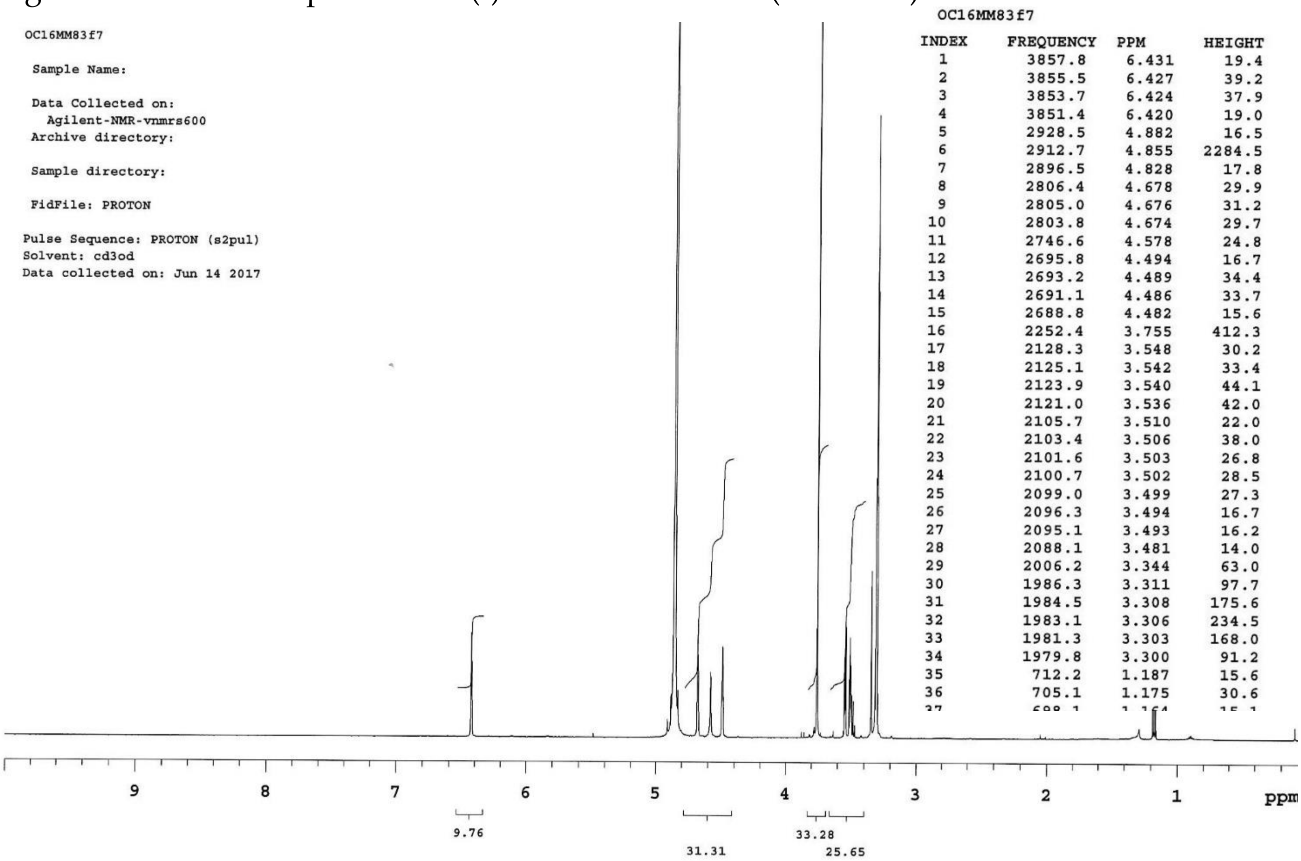

Figure S29. ^{13}C-NMR spectrum of (-)-**12** in methanol-d4 (150 MHz)

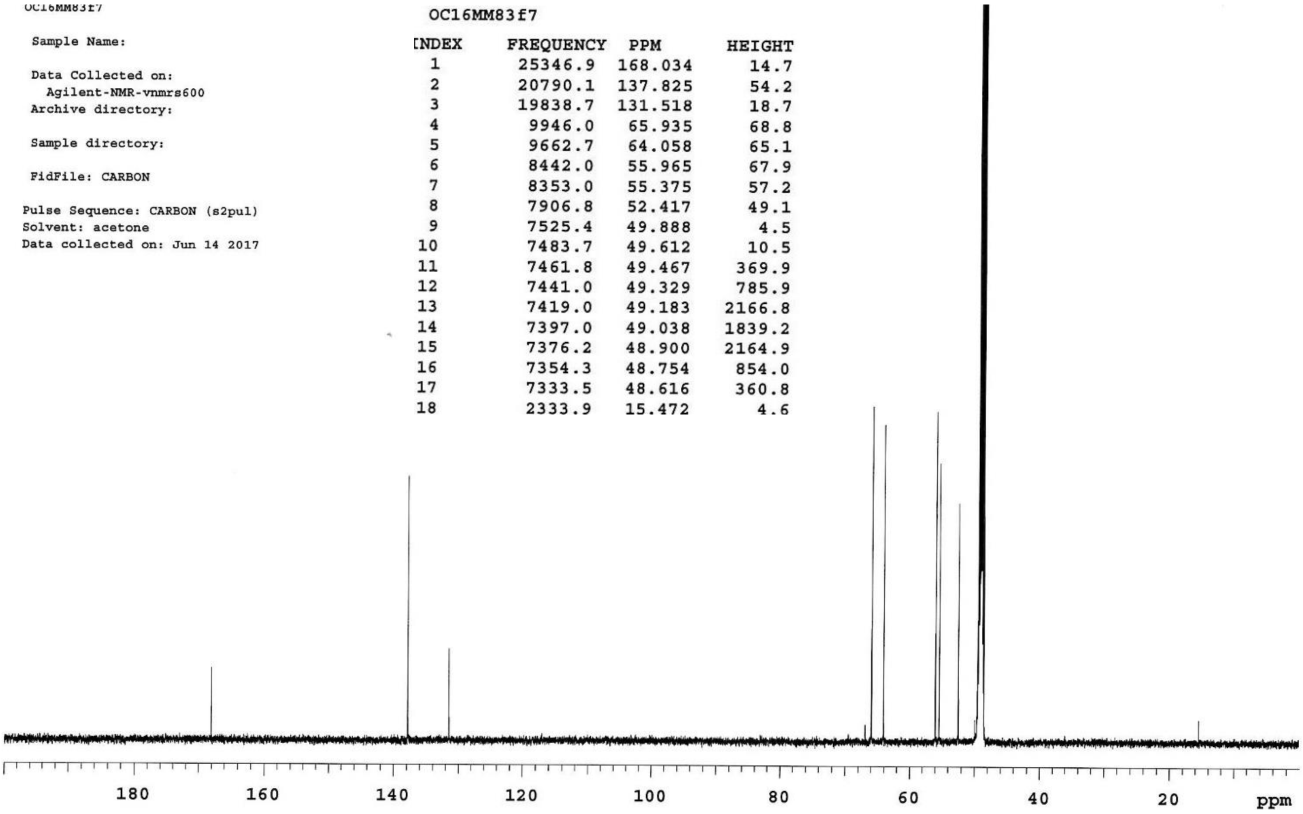

Figure S30. HMBC-NMR spectrum of (-)-**12** in methanol-d4 (600 MHz)

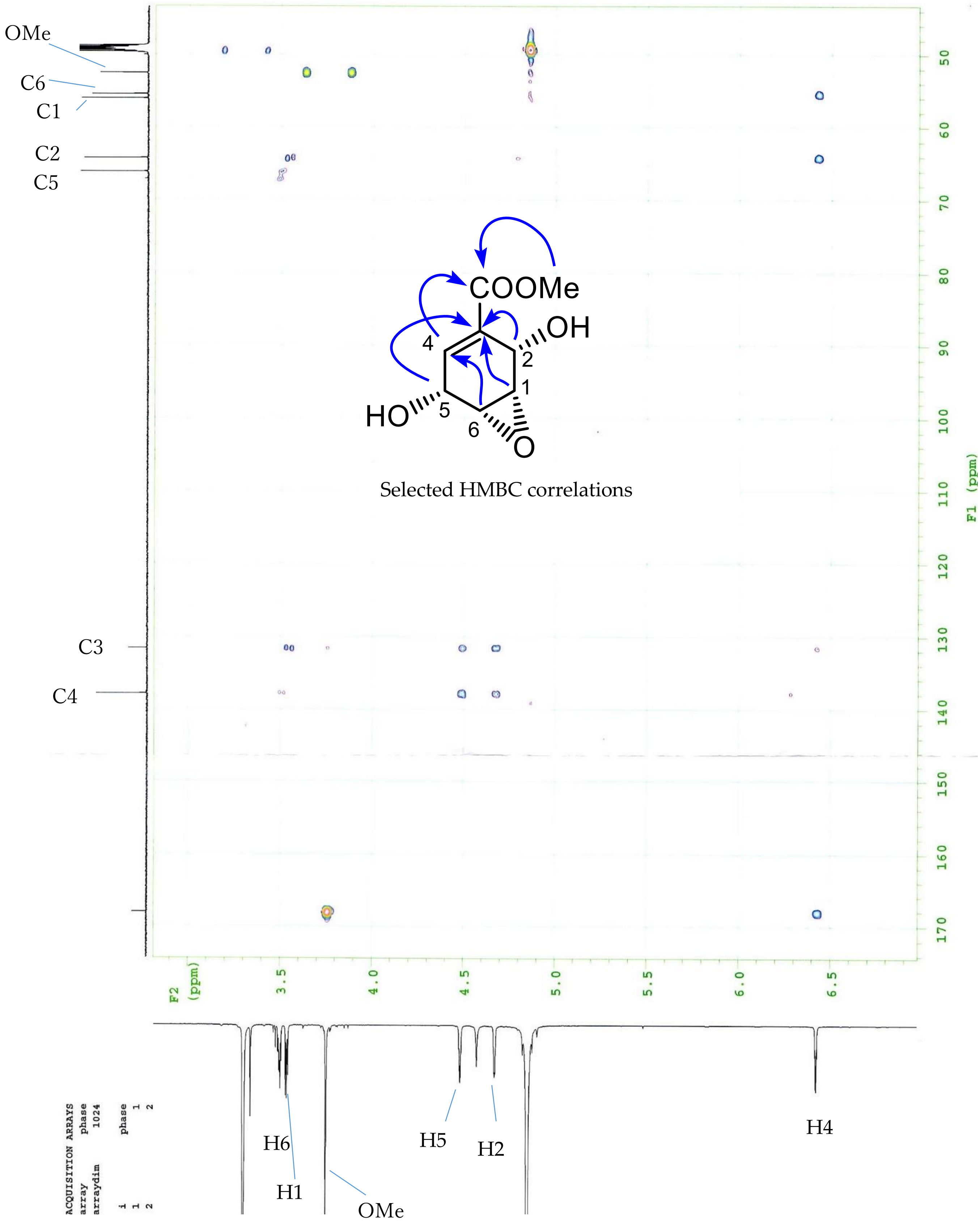

Figure S31. ^{1}H-NMR spectrum of (-)-**7** in methanol-d$_4$ (600 MHz)

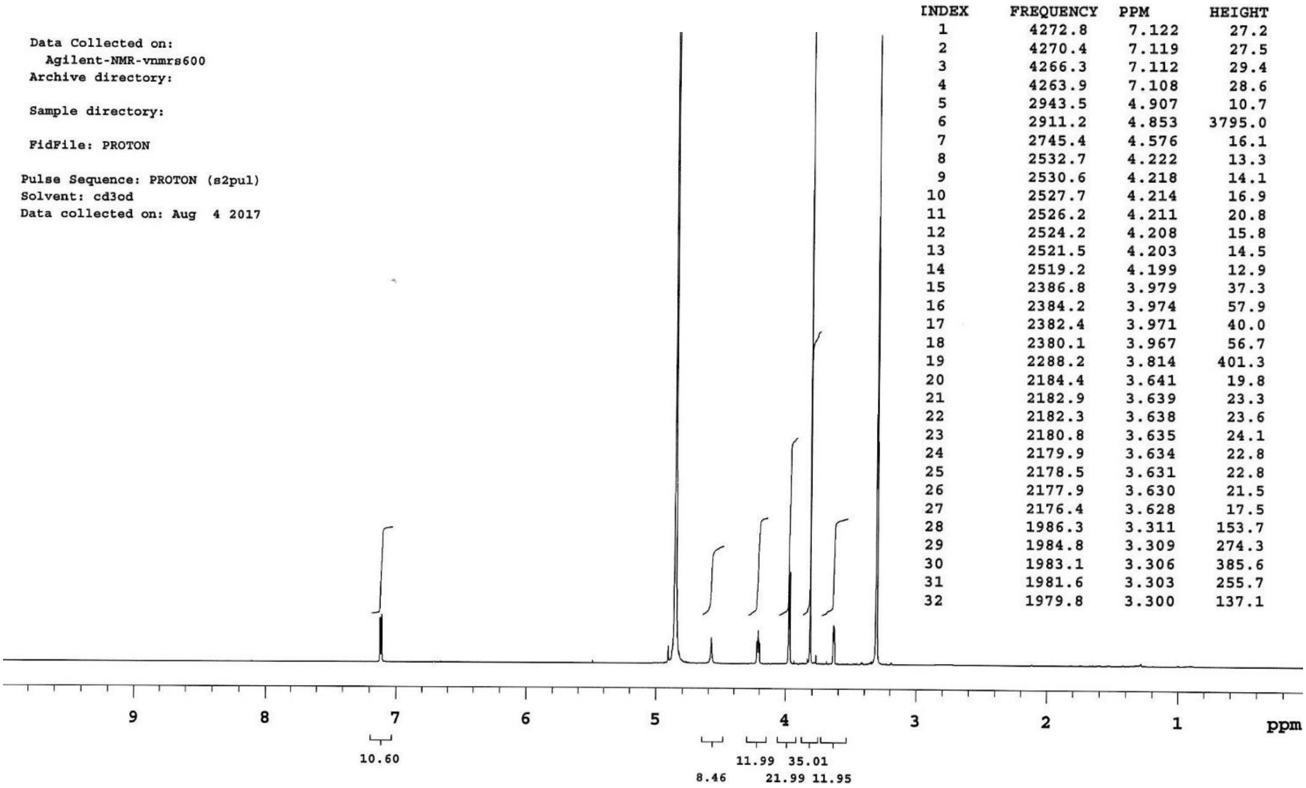

INDEX	FREQUENCY	PPM	HEIGHT
1	4272.8	7.122	27.2
2	4270.4	7.119	27.5
3	4266.3	7.112	29.4
4	4263.9	7.108	28.6
5	2943.5	4.907	10.7
6	2911.2	4.853	3795.0
7	2745.4	4.576	16.1
8	2532.7	4.222	13.3
9	2530.6	4.218	14.1
10	2527.7	4.214	16.9
11	2526.2	4.211	20.8
12	2524.2	4.208	15.8
13	2521.5	4.203	14.5
14	2519.2	4.199	12.9
15	2386.8	3.979	37.3
16	2384.2	3.974	57.9
17	2382.4	3.971	40.0
18	2380.1	3.967	56.7
19	2288.2	3.814	401.3
20	2184.4	3.641	19.8
21	2182.9	3.639	23.3
22	2182.3	3.638	23.6
23	2180.8	3.635	24.1
24	2179.9	3.634	22.8
25	2178.5	3.631	22.8
26	2177.9	3.630	21.5
27	2176.4	3.628	17.5
28	1986.3	3.311	153.7
29	1984.8	3.309	274.3
30	1983.1	3.306	385.6
31	1981.6	3.303	255.7
32	1979.8	3.300	137.1

Figure S32. ^{13}C-NMR spectrum of (-)-**7** in methanol-d$_4$ (150 MHz)

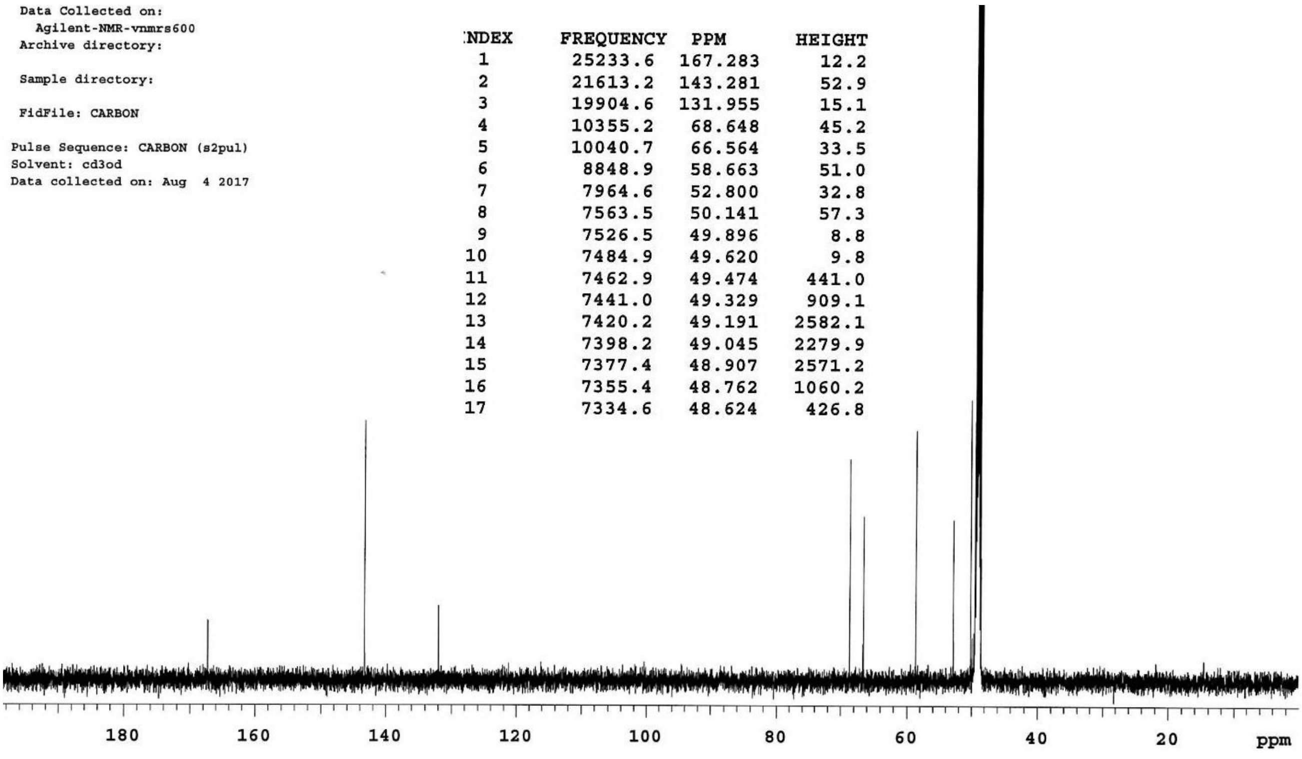

INDEX	FREQUENCY	PPM	HEIGHT
1	25233.6	167.283	12.2
2	21613.2	143.281	52.9
3	19904.6	131.955	15.1
4	10355.2	68.648	45.2
5	10040.7	66.564	33.5
6	8848.9	58.663	51.0
7	7964.6	52.800	32.8
8	7563.5	50.141	57.3
9	7526.5	49.896	8.8
10	7484.9	49.620	9.8
11	7462.9	49.474	441.0
12	7441.0	49.329	909.1
13	7420.2	49.191	2582.1
14	7398.2	49.045	2279.9
15	7377.4	48.907	2571.2
16	7355.4	48.762	1060.2
17	7334.6	48.624	426.8

Figure S33. ^{1}H-NMR spectrum of (+)-**7** in methanol-d$_4$ (600 MHz)

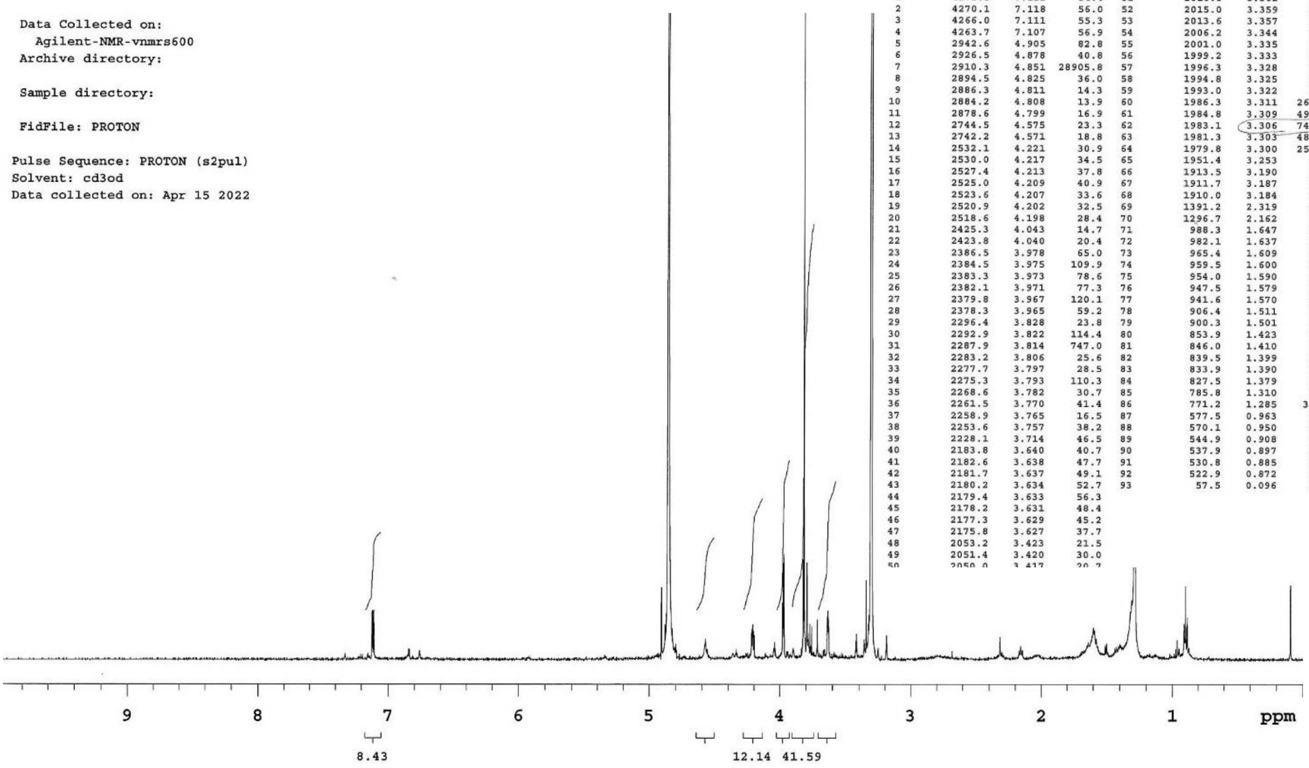

INDEX	FREQUENCY	PPM	HEIGHT	INDEX	FREQUENCY	PPM	HEIGHT
1	4272.5	7.122	54.4	51	2016.8	3.362	17.2
2	4270.1	7.118	56.0	52	2015.0	3.359	23.1
3	4266.0	7.111	55.3	53	2013.6	3.357	19.2
4	4263.7	7.107	56.9	54	2006.2	3.344	90.5
5	2942.6	4.905	82.8	55	2001.0	3.335	19.9
6	2926.5	4.878	40.8	56	1999.2	3.333	22.8
7	2910.3	4.851	28905.8	57	1996.3	3.328	41.4
8	2894.5	4.825	36.0	58	1994.8	3.325	42.8
9	2886.3	4.811	14.3	59	1993.0	3.322	51.1
10	2884.2	4.808	13.9	60	1986.3	3.311	2690.3
11	2878.6	4.799	16.9	61	1984.8	3.309	4945.8
12	2744.5	4.575	23.3	62	1983.1	3.306	7487.2
13	2742.2	4.571	18.8	63	1981.3	3.303	4856.4
14	2532.1	4.221	30.9	64	1979.8	3.300	2573.7
15	2530.0	4.217	34.5	65	1951.4	3.253	13.9
16	2527.4	4.213	37.8	66	1913.5	3.190	18.9
17	2525.0	4.209	40.9	67	1911.7	3.187	27.9
18	2523.6	4.207	33.6	68	1910.0	3.184	18.0
19	2520.9	4.202	32.5	69	1391.2	2.319	26.8
20	2518.6	4.198	28.4	70	1296.7	2.162	15.6
21	2425.3	4.043	14.7	71	988.3	1.647	16.0
22	2423.8	4.040	20.4	72	982.1	1.637	19.9
23	2386.5	3.978	65.0	73	965.4	1.609	28.5
24	2384.5	3.975	109.9	74	959.5	1.600	37.0
25	2383.3	3.973	78.6	75	954.0	1.590	33.8
26	2382.1	3.971	77.3	76	947.5	1.579	24.2
27	2379.8	3.967	120.1	77	941.6	1.570	16.0
28	2378.3	3.965	59.2	78	906.4	1.511	15.8
29	2296.4	3.828	23.8	79	900.3	1.501	17.0
30	2292.9	3.822	114.4	80	853.9	1.423	14.8
31	2287.9	3.814	747.0	81	846.0	1.410	15.5
32	2283.2	3.806	25.6	82	839.5	1.399	16.9
33	2277.7	3.797	28.5	83	833.9	1.390	16.6
34	2275.3	3.793	110.3	84	827.5	1.379	15.0
35	2268.6	3.782	30.7	85	785.8	1.310	67.4
36	2261.5	3.770	41.4	86	771.2	1.285	394.1
37	2258.9	3.765	16.5	87	577.5	0.963	22.7
38	2253.6	3.757	38.2	88	570.1	0.950	13.9
39	2228.1	3.714	46.5	89	544.9	0.908	41.9
40	2183.8	3.640	40.7	90	537.9	0.897	84.2
41	2182.6	3.638	47.7	91	530.8	0.885	48.5
42	2181.7	3.637	49.1	92	522.9	0.872	14.0
43	2180.2	3.634	52.7	93	57.5	0.096	85.1
44	2179.4	3.633	56.3				
45	2178.2	3.631	48.4				
46	2177.3	3.629	45.2				
47	2175.8	3.627	37.7				
48	2053.2	3.423	21.5				
49	2051.4	3.420	30.0				
50	2050.0	3.417	20.7				

Figure S34. ^{13}C-NMR spectrum of (+)-**7** in methanol-d$_4$ (150 MHz)

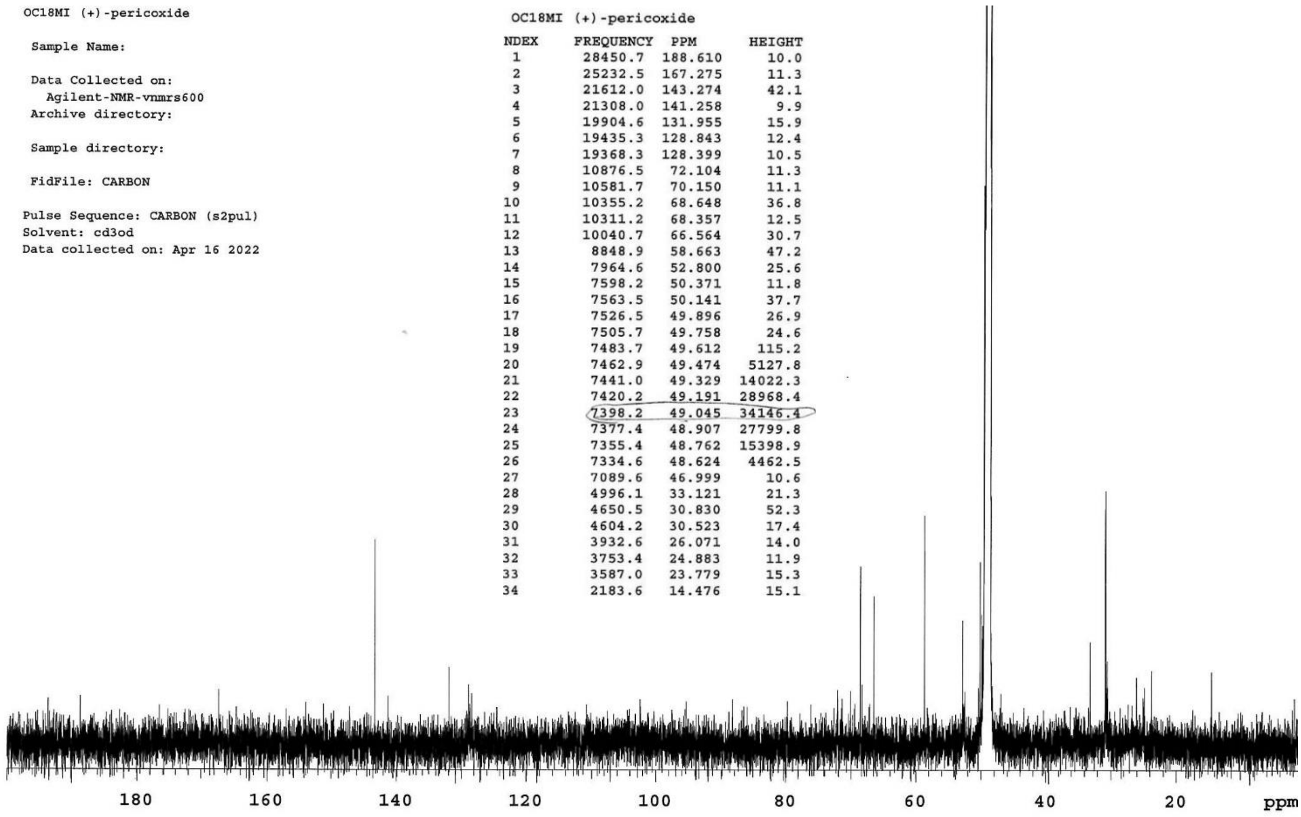

NDEX	FREQUENCY	PPM	HEIGHT
1	28450.7	188.610	10.0
2	25232.5	167.275	11.3
3	21612.0	143.274	42.1
4	21308.0	141.258	9.9
5	19904.6	131.955	15.9
6	19435.3	128.843	12.4
7	19368.3	128.399	10.5
8	10876.5	72.104	11.3
9	10581.7	70.150	11.1
10	10355.2	68.648	36.8
11	10311.2	68.357	12.5
12	10040.7	66.564	30.7
13	8848.9	58.663	47.2
14	7964.6	52.800	25.6
15	7598.2	50.371	11.8
16	7563.5	50.141	37.7
17	7526.5	49.896	26.9
18	7505.7	49.758	24.6
19	7483.7	49.612	115.2
20	7462.9	49.474	5127.8
21	7441.0	49.329	14022.3
22	7420.2	49.191	28968.4
23	7398.2	49.045	34146.4
24	7377.4	48.907	27799.8
25	7355.4	48.762	15398.9
26	7334.6	48.624	4462.5
27	7089.6	46.999	10.6
28	4996.1	33.121	21.3
29	4650.5	30.830	52.3
30	4604.2	30.523	17.4
31	3932.6	26.071	14.0
32	3753.4	24.883	11.9
33	3587.0	23.779	15.3
34	2183.6	14.476	15.1

2. Table S1. *Deprotection of (-)-**10**$_{Br}$ with Dowex® 50WX8-H*

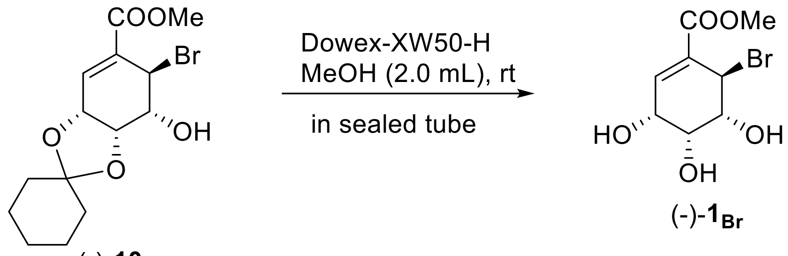

(-)-**10**$_{Br}$
ca. 40 mg (0.12 mmol)

(-)-**1**$_{Br}$

entry	Dowex XW-H	temperature	time (h)	**1**$_{Br}$, yield (%)
1	108 mg	80 °C (MW)*	1	32
2	102 mg	0 °C	86	47
3	108 mg	rt	20	55
4	101 mg	rt	40	73
5	103 mg	rt	56	87
6	51.0 mg	rt	64	63

General procedure: entry 4 (same as described in text, experimental section); To a solution of (-)-**10**$_{Br}$ (41.3 mg, 0.12 mmol) in MeOH (2.0 mL) was added Dowex® 50WX8-H (102.5 mg). The reaction mixture was stirred for 56 h at room temperature. The reaction mixture was filtered. The filtrate, which was condensed under reduced pressure to give a crude residue which was purified with silica gel column chromatography (eluent; CH_2Cl_2: MeOH = 95: 5) to afford **(-)-1**$_{Br}$ (27.6 mg, 87%) with recovery of the starting (-)-**10**$_{Br}$ (5.5 mg, 13%).

*Results suggested that reduced amount of resin decreased chemical yield of desired **1**$_{Br}$ (entry 6) and optimum reaction time was 56 h (entries 3-5).
